# Disease Burden and Pharmacological Treatment Patterns in Children and Adults With Phenylketonuria: A Real‐World Matched Cohort Study

**DOI:** 10.1002/jimd.70194

**Published:** 2026-04-27

**Authors:** Nicola Longo, Barbara K. Burton, Shailja Vaghela, Fan Mu, Aolin Wang, Jess Smith, Emily Reichert, Ryan B. Simpson, Vanja Sikirica, Sue Perera

**Affiliations:** ^1^ Department of Human Genetics, Division of Clinical Genetics University of California Los Angeles California USA; ^2^ Northwestern University Feinberg School of Medicine Chicago Illinois USA; ^3^ Moderna, Inc. Cambridge Massachusetts USA; ^4^ HealthEcon Consulting, Inc. Ancaster Ontario Canada; ^5^ Analysis Group Boston Massachusetts USA; ^6^ Moderna, Inc. Princeton New Jersey USA

**Keywords:** claims‐based retrospective studies, clinical burden, healthcare resource utilization, phenylalanine, phenylketonuria, real‐world evidence

## Abstract

Phenylketonuria (PKU) is caused by defective catabolism of phenylalanine (Phe), resulting in Phe accumulation and subsequent neurocognitive impairment. This retrospective study used a large United States claims database linked to laboratory records (6/2018–05/2023) to compare comorbidities, healthcare resource utilization, and healthcare costs between individuals with PKU and matched controls, stratified by age and Phe level, as well as describe pharmacological treatment patterns among individuals with PKU. The study included 5729 and 2648 matched pairs aged ≥ 12 and < 12 years, respectively (mean age: 31 and 3 years; 60% and 47% female). Individuals aged ≥ 12 years with PKU had significantly higher prevalence of neuropsychiatric and cognitive comorbidities than controls, including intellectual or developmental disabilities (prevalence difference: 5.2%), fatigue and malaise (4.9%), and anxiety (4.2%; all *p* < 0.05), along with other comorbidities across multiple organ systems. Individuals aged ≥ 12 years with PKU also accessed the healthcare system more than controls (39% more hospitalizations, 18% more emergency room visits, and 25% more outpatient visits) and incurred higher medical and pharmacy costs ($2869 and $9075, respectively; *p* < 0.05). Individuals aged < 12 years similarly had higher burden than controls, but only for certain outcomes and at smaller magnitudes. Individuals with Phe ≥ 600 μmol/L showed a greater incremental burden for certain comorbidities, such as intellectual or developmental disabilities, compared to individuals with Phe < 600 μmol/L. These findings demonstrate the substantial burden of PKU across age and Phe level strata, notably in those aged ≥ 12 years, highlighting the need for improved treatments.

## 1 Introduction

Phenylketonuria (PKU) is a rare inherited metabolic disorder caused by defective phenylalanine hydroxylase (PAH), the enzyme converting phenylalanine (Phe) to tyrosine, leading to elevated Phe levels [1]. It has an estimated prevalence of 1 in 25 000 newborns in the United States (US) [1, 2], where universal newborn screening for PKU has been ongoing since the 1960s [3]. PKU, if untreated, can lead to intellectual disabilities, developmental delays, and neurological disorders [1, 2, 4, 5, 6, 7, 8, 9]. In addition to this clinical burden, there is evidence that individuals with PKU have a high economic burden and experience higher rates of healthcare utilization (HCRU) and healthcare costs compared to those without the disease [10, 11, 12, 13, 14].

Treatment guidelines recommend initiation of therapy immediately after birth, with a focus on lowering Phe levels to recommended ranges (120–360 μmol/L) [5, 8, 15]. A common management approach is life‐long dietary restriction of protein, in addition to medical foods that are Phe‐free, to reduce blood Phe levels [16, 17, 18]. Although this approach is effective [19], it can be time‐consuming, onerous for caregivers, and financially burdensome [11, 20, 21, 22, 23]. Thus, adherence rates are high in infancy and childhood, but typically decline during adolescence and adulthood [4, 5, 7].

Three medications have been introduced to complement dietary Phe management. These include sapropterin dihydrochloride and sepiapterin, approved for use in patients aged 1 month or older by the US Food and Drug Administration (FDA) in 2007 and 2025, respectively [24, 25], and pegvaliase, FDA‐approved for adults in 2018 [26]. Despite the available treatment options, individuals with PKU continue to experience a high clinical burden related to neuropsychiatric and cognitive symptoms (e.g., intellectual disabilities, executive dysfunction) [17, 18, 27, 28, 29, 30, 31, 32, 33, 34, 35, 36, 37, 38, 39]. In adulthood, individuals with PKU also exhibit high rates of comorbidities across other organ systems, encompassing but not limited to, musculoskeletal (e.g., osteoarthritis), metabolic and endocrine (e.g., lipoprotein metabolism disorders), cardiorenal (e.g., chronic ischemic heart disease), digestive (e.g., pancreatitis), and dermatological (e.g., psoriasis) conditions [14, 35, 37, 40, 41, 42].

Despite the established burden of the disease, to our knowledge, there are no contemporary real‐world studies that comprehensively assess the clinical and economic burden of PKU stratified by age (separating children from adolescents) or clinically‐relevant Phe level categorizations. Given the more recent drug approvals, there is also a need to characterize pharmacological treatment use and patterns among individuals with PKU. To address these gaps, this study aimed to comprehensively describe and compare the clinical burden, HCRU, and healthcare costs of individuals with PKU to that of matched controls without PKU, overall and stratified by age group and Phe level. Additionally, this study described pharmacological treatment patterns of individuals with PKU by age.

## 2 Methods

### 2.1 Study Design

A retrospective matched observational cohort study was performed among individuals with PKU and matched individuals without PKU (i.e., controls). Closed medical and pharmacy claims data from HealthVerity US health insurance claims data (June 2018 to May 2023), containing information from over 150 unique payers for over 115 million patients, were linked to laboratory assessment records (Quest Diagnostics). The data were de‐identified and compliant with requirements of the Health Insurance Portability and Accountability Act (HIPAA) of 1996; therefore, no Institutional Review Board waiver of informed consent approval or exemption was required, as per article 45 §CFR 164.514(e).

### 2.2 Study Population

#### 2.2.1 Eligibility Criteria

The data cut included records for individuals with PKU and individuals without PKU based on crude matching (1:10) on age, sex, and US region, where possible. The PKU cohort included individuals with a diagnosis of classical PKU (International Classification of Diseases, 10th Revision, Clinical Modification [ICD‐10‐CM]: E70.0) and no suspected tetrahydrobiopterin (BH4) deficiency (ICD‐10‐CM: E70.1, with a pharmacy fill of levodopa and/or carbidopa) at any time. The non‐PKU cohort included individuals with no diagnosis of classical PKU nor BH4 deficiency at any time.

The index date was defined as the first day of the first eligible continuous enrollment period. The follow‐up period was defined as the time from the index date until the earliest of (1) end of the data cut or (2) end of medical or pharmacy eligibility. Both cohorts were required to have continuous medical and pharmacy enrollment for ≥ 12 months and no missing or unknown matching variables.

#### 2.2.2 Matching

Matching of individuals with PKU and controls was conducted on a 1:1 ratio based on age at index, sex, US region, index year category (2018–2019 or 2020–2022), and medical and pharmacy insurance plan type. Age at index was exactly matched for individuals aged < 18 years and matched with a caliper of +/− 1 year for individuals aged ≥ 18 years. Additionally, cohorts were partially matched on follow‐up time, where the minimum follow‐up time for a control was the follow‐up time of the matched individual with PKU, rounded down to the nearest 6‐month increment (up to a maximum of 30 months). For example, for individuals with PKU with 20 months of follow‐up, controls were required to have at least 18 (3 × 6) months of follow‐up. After matching, the follow‐up time of the individual with PKU or the control was truncated to the shorter follow‐up time of the matched pair to ensure identical follow‐up duration.

### 2.3 Subpopulations

#### 2.3.1 Lab Subpopulation

The ‘lab subpopulation’ was identified from the post‐matching sample. This subpopulation included individuals with PKU who had at least one Phe lab assessment result during the follow‐up period, and their matched controls, who may or may not have had lab assessment data (i.e., lab assessment data was not required for controls).

#### 2.3.2 Treatment Subpopulation

The “treatment subpopulation” included individuals with PKU from the post‐matching sample who had at least one pharmacy fill of sapropterin dihydrochloride or pegvaliase during the follow‐up period. Matched controls were not included for this subpopulation. The index date was redefined as the date of first observed treatment initiation. Treated individuals with PKU were further required to have continuous medical and pharmacy enrollment for ≥ 24 months to allow for observation of treatment patterns. Information on diet therapy was not available, and therefore, the treatment subpopulation focuses only on those receiving pharmacological therapy for PKU.

### 2.4 Variables and Outcomes

#### 2.4.1 Overall Population

Demographic characteristics were assessed as of the index date, or the closest date to index if data were missing on the index date. Outcomes assessed during the follow‐up period included a comprehensive list of PKU‐related comorbidities across multiple organ systems, all‐cause HCRU, and all‐cause healthcare costs.

All‐cause HCRU included inpatient, neonatal intensive care unit/intensive care unit (NICU/ICU), emergency department (ED), and outpatient visits. All‐cause costs included medical (i.e., inpatient, ED, outpatient, and other) and pharmacy costs. Costs were limited to those available in the medical and pharmacy claims data, and therefore, costs of over‐the‐counter medications and dietary management of PKU were omitted.

#### 2.4.2 Lab Subpopulation

All outcomes assessed in the overall population were repeated among the lab subpopulation. Additionally, Phe and tyrosine (Tyr) levels were assessed during the follow‐up period.

#### 2.4.3 Treatment Subpopulation

Demographics assessed for the overall population were repeated among the treatment subpopulation using the updated index date. Pharmacological treatment patterns were described during the follow‐up period. Treatment discontinuation was defined as the initiation of a new treatment from the index treatment or the first gap while on the index treatment. A treatment gap of > 90 days for sapropterin dihydrochloride or > 30 days for pegvaliase from the last day of supply was considered a discontinuation. Discontinuation definitions differed by therapy to reflect expected dispensing patterns: a longer gap was used for sapropterin dihydrochloride, an oral medication with variable refill intervals, relative to the injection‐based pegvaliase.

### 2.5 Subgroups, Stratifications, and Sensitivity Analysis

The results were assessed overall and stratified by age (< 12 and ≥ 12 years). For the lab subpopulation, the results were stratified by Phe level (≥ 600 and < 600 μmol/L). If more than 1 Phe lab assessment was available for an individual during the follow‐up period, the maximum value was used. For the treatment subpopulation, the results were stratified by age (< 12 and ≥ 12 years) and first observed treatment (sapropterin dihydrochloride or pegvaliase). To evaluate robustness to birth cohort differences related to newborn screening implementation, we repeated primary matched comparisons of comorbidities in the subset of individuals in the ≥ 12‐year cohort with birth year ≥ 1965.

### 2.6 Statistical Analyses

Variables were summarized descriptively using means and standard deviations (SDs) for continuous variables and counts and percentages for categorical variables. Prevalence difference (PD) was used to assess the incremental clinical burden associated with PKU versus controls. Comparisons were made between individuals with PKU and matched controls using McNemar's tests.

PKU‐related comorbidities were evaluated across multiple organ systems. The main text presents a clinically selected subset from three organ systems (neuropsychiatric and cognitive, musculoskeletal, and metabolic and endocrine) meeting two criteria: prevalence > 5% in PKU or controls and a statistically significant difference in the overall population. The full list of comorbidities is included in Table S1. Comorbidities are listed by descending order of PD in the overall population.

All‐cause HCRU outcomes were described and compared between individuals with PKU and matched controls using generalized estimating equations. Outcomes were reported as rates per person‐year (PPY), calculated as the number of events divided by the person‐years, and incidence rate ratios (IRRs) with corresponding 95% confidence intervals (CIs). Costs were inflated to 2023 US dollars (USD). Annualized all‐cause healthcare costs were compared using Wilcoxon signed‐rank tests.

Statistical tests were conducted in R version 4.2.2. A *p*‐value of < 0.05 was considered statistically significant.

## 3 Results

### 3.1 Overall Population

#### 3.1.1 Sample Selection

**FIGURE 1:**
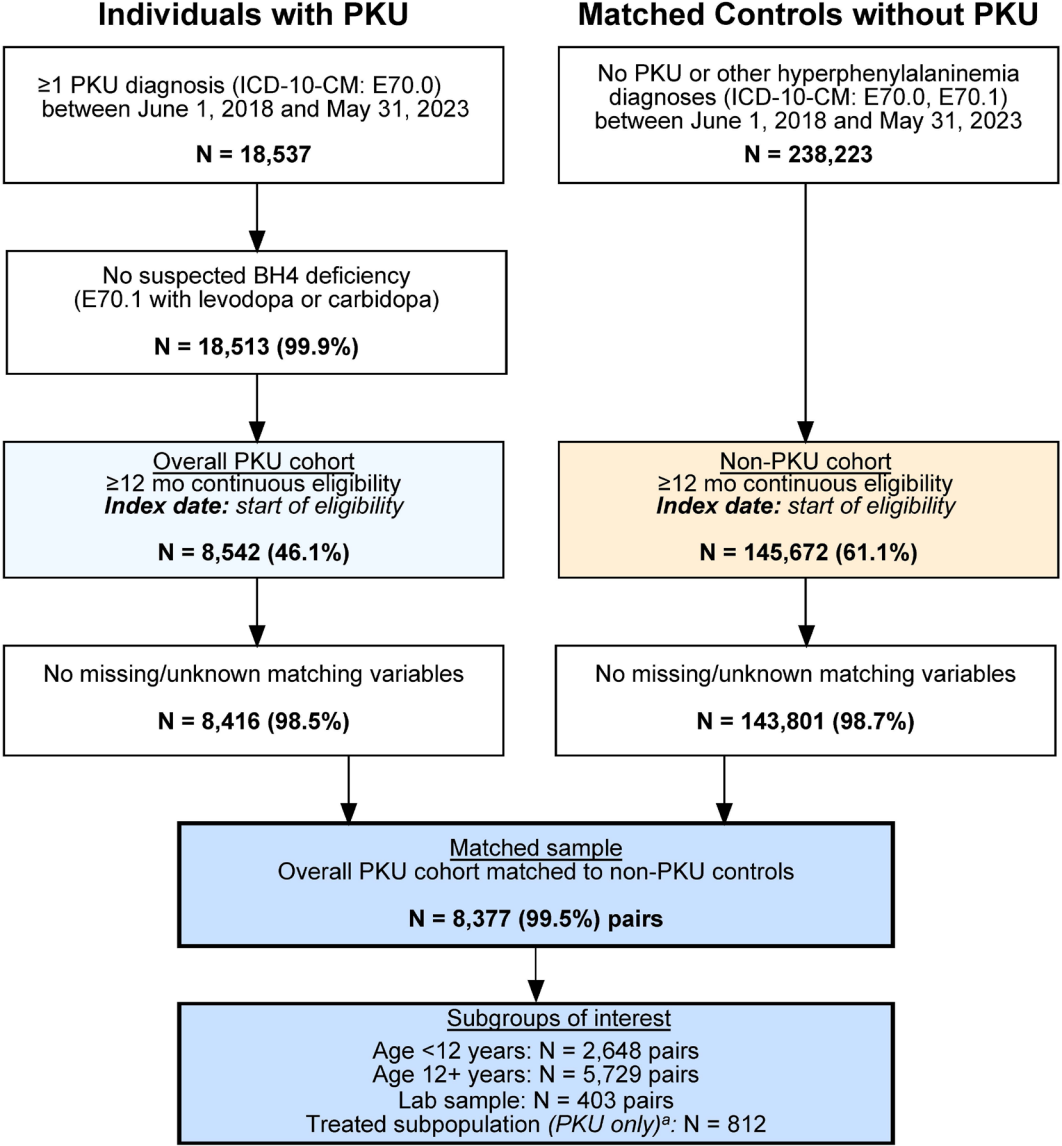
Sample selection. *Note:* For the treated subpopulation, the index date was redefined as the date of first observed treatment initiation. ≥ 24 months of continuous eligibility was required for the treated subpopulation. Abbreviations: BH4, tetrahydrobiopterin; ICD‐10‐CM, International Classification of Diseases, 10th edition—Clinical Modification; PKU, phenylketonuria.

The cohort selection process is shown in Figure 1. After matching, there were 8377 individuals with PKU and their matched controls, with 2648 matched pairs aged < 12 years and 5729 matched pairs aged ≥ 12 years.

#### 3.1.2 Demographic Characteristics

The mean age was 31.3 (SD: 26.1) years, with 56.2% female and 53.7% on Medicaid managed care insurance (Table 1). The mean ages were 2.9 (SD: 3.3) and 44.4 (21.0) years for those aged < 12 and ≥ 12 years, respectively. Compared to the < 12 years age group, the proportion of females was higher in the ≥ 12 years age group (60.4% vs. 47.1%) and fewer were covered by Medicaid managed care (42.9% vs. 77.0%).

**TABLE 1:** Demographic characteristics for individuals with PKU matched to non‐PKU controls.

	Overall population			Lab subpopulation		
	Overall	< 12 years	≥ 12 years	Overall	Phe < 600 μmol/L	Phe ≥ 600 μmol/L
	*N* = 8377 pairs	*N* = 2648 pairs	*N* = 5729 pairs	*N* = 403 pairs	*N* = 220 pairs	*N* = 183 pairs
Age^a^, mean ± SD (years)	31.3 ± 26.1	2.9 ± 3.3	44.4 ± 21.0	18.9 ± 15.7	14.1 ± 13.1	24.7 ± 16.7
Female, *n* (%)	4704 (56.2%)	1246 (47.1%)	3458 (60.4%)	212 (52.6%)	115 (52.3%)	97 (53.0%)
Region, *n* (%)						
Northeast	1729 (20.6%)	256 (9.7%)	1473 (25.7%)	51 (12.7%)	27 (12.3%)	24 (13.1%)
Midwest	1297 (15.5%)	437 (16.5%)	860 (15.0%)	60 (14.9%)	38 (17.3%)	22 (12.0%)
South	2959 (35.3%)	1444 (54.5%)	1515 (26.4%)	159 (39.5%)	88 (40.0%)	71 (38.8%)
West	2123 (25.3%)	506 (19.1%)	1617 (28.2%)	129 (32.0%)	67 (30.5%)	62 (33.9%)
Other	269 (3.2%)	5 (0.2%)	264 (4.6%)	4 (1.0%)	0 (0.0%)	4 (2.2%)
Medical insurance type, *n* (%)						
Commercial	2854 (34.1%)	607 (22.9%)	2247 (39.2%)	138 (34.2%)	73 (33.2%)	65 (35.5%)
Medicaid	4496 (53.7%)	2040 (77.0%)	2456 (42.9%)	258 (64.0%)	146 (66.4%)	112 (61.2%)
Medicare Advantage	1027 (12.3%)	1 (0.0%)	1026 (17.9%)	7 (1.7%)	1 (0.5%)	6 (3.3%)
Index year (2018–2019), *n* (%)	6072 (72.5%)	1379 (52.1%)	4693 (81.9%)	326 (80.9%)	175 (79.5%)	151 (82.5%)

Abbreviations: Phe, phenylalanine; PKU, phenylketonuria; SD, standard deviation.

^a^ Patient age, rounded down to the nearest year, was calculated on the index date using an imputed date of birth of July 1 of the patient's year of birth. For patients with an index date prior to July 1 in the same year they were born, patient age at index was assigned as 0 years.

#### 3.1.3 Clinical Burden

**FIGURE 2:**
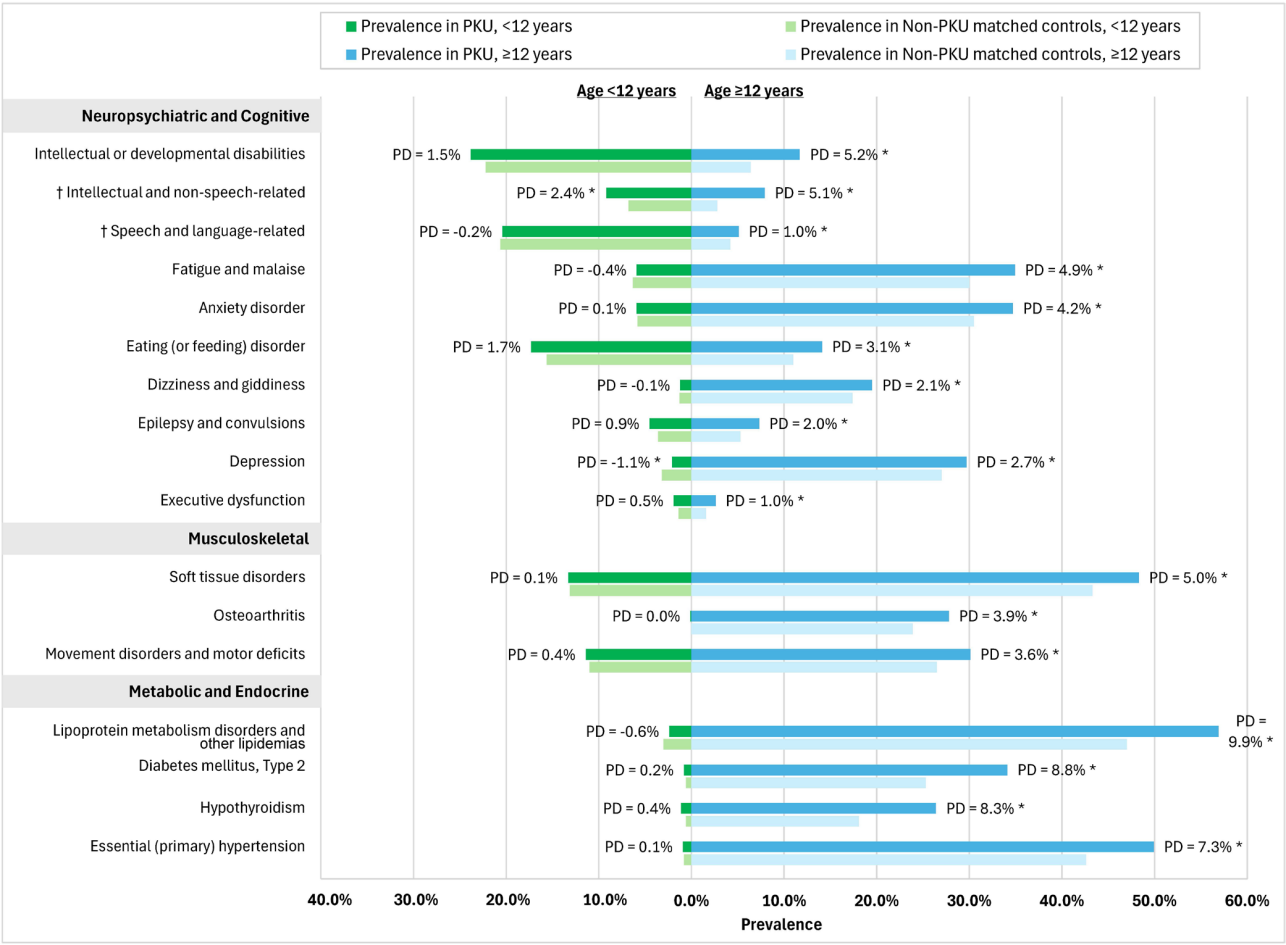
Prevalence of select comorbidities in individuals with PKU compared to Non‐PKU controls, by age. *Note:* Select comorbidities were arranged based on descending order of PD from the overall cohort within each organ system. Among the comprehensive list of PKU‐related comorbidities, a clinically selected subset from three organ systems (neuropsychiatric and cognitive, musculoskeletal, and metabolic and endocrine) meeting two criteria: Prevalence > 5% in PKU or controls and a statistically significant difference in the overall population, are presented. Results for the full list of comorbidities are included in the Supplementary Tables. Abbreviations: PD, prevalence difference; PKU, phenylketonuria. *p < 0.05. ^†^These comorbidities are subcategories of intellectual or developmental disabilities.

Among individuals aged ≥ 12 years, individuals with PKU had statistically higher (*p* < 0.001) prevalence of various comorbidities across multiple organ systems compared to their matched controls (Figure 2). Within the neuropsychiatric and cognitive domain, the comorbidities with the highest PD were intellectual or developmental disabilities (IDDs; PKU vs. non‐PKU: 11.7% vs. 6.4% [PD: 5.2%]), fatigue and malaise (34.9% vs. 30.0% [4.9%]), and anxiety disorder (34.7% vs. 30.5% [4.2%]). Across other organ systems, comorbidities with notable PD included lipoprotein metabolism disorders and other lipidemias (56.9% vs. 47.0% [PD: 9.9%]), Type 2 diabetes (34.1% vs. 25.3% [8.8%]), and hypothyroidism (26.4% vs. 18.1% [8.3%]). For individuals aged < 12 years, the only comorbidity observed to be significantly elevated (*p* < 0.01) in individuals with PKU compared to non‐PKU matched controls was intellectual and non‐speech‐related developmental disabilities (9.2% vs. 6.8% [PD: 2.4%]).

In the sensitivity analyses restricted to individuals with birth year ≥ 1965, the pattern of comorbidity differences remained largely consistent with that of the main analyses. Statistically significant prevalence differences (all *p* < 0.05) persisted across multiple organ systems, including IDDs (PD: 4.9%), fatigue and malaise (PD: 3.6%), anxiety disorders (PD: 3.6%), lipoprotein metabolism disorders and other lipidemias (PD: 9.1%), Type 2 diabetes (PD: 6.3%), and hypothyroidism (PD: 5.8%).

#### 3.1.4 Economic Burden

##### 3.1.4.1 Healthcare Resource Utilization

**FIGURE 3:**
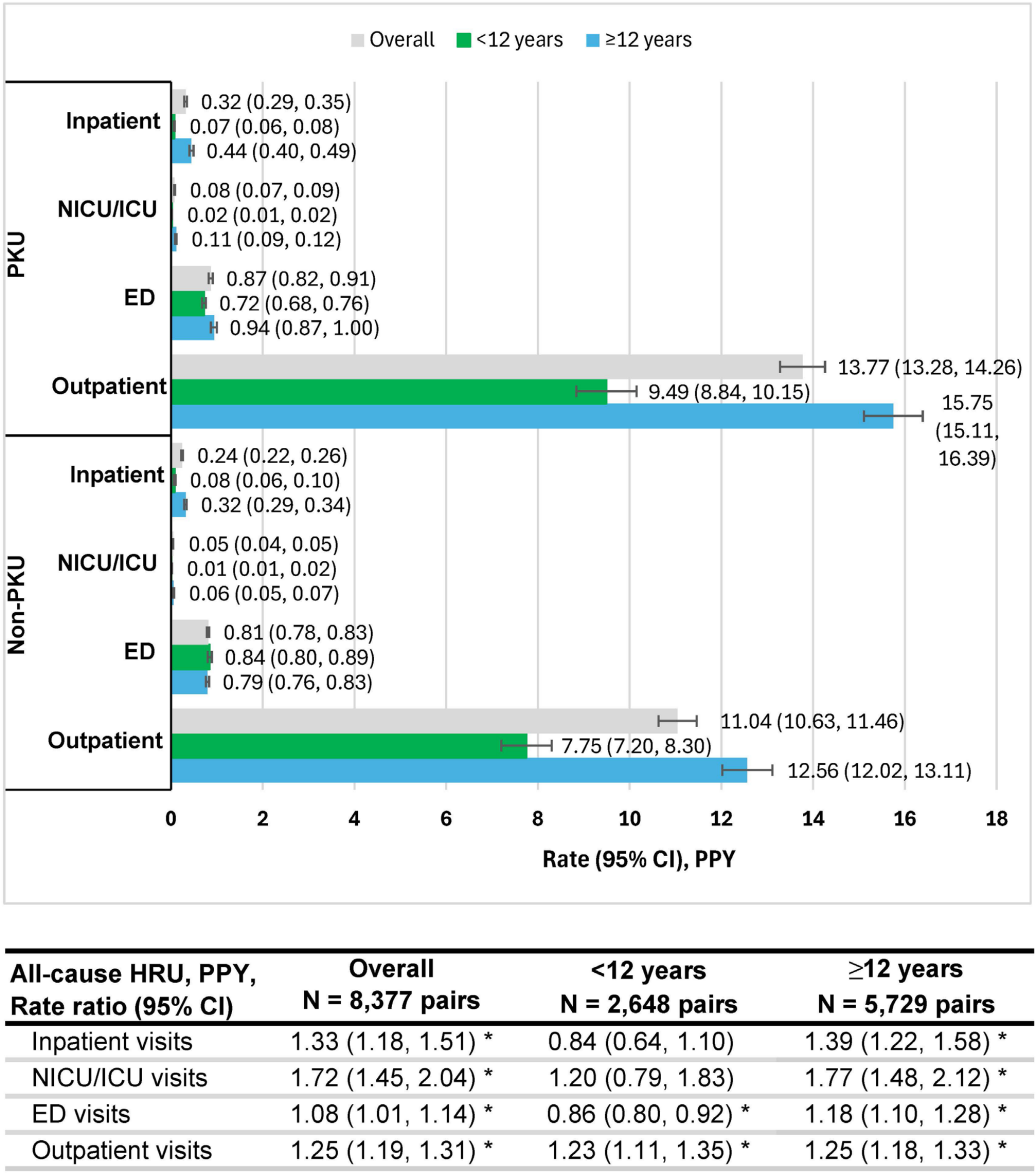
Healthcare resource utilization in individuals with PKU compared to Non‐PKU controls. *Note:* Rate ratios and 95% CIs were calculated using a generalized estimating equations model with data clustered by matching ID. The model used a log link function (outcome was assumed to be Poisson distributed), with an exchangeable correlation structure. HCRU settings were flagged at the day level based on place of service codes, type of bill codes, revenue codes, and Current Procedural Terminology/Healthcare Common Procedure Coding System codes. Encounter periods were created based on the presence of flags in consecutive days, except for outpatient stays for which each day was counted as an individual visit. ED and outpatient stays that overlapped with an inpatient visit were removed. Four ED stays were removed from the data due to long length of stays greater than 100 days. Abbreviations: CI, confidence interval; ED, emergency department; HCRU, healthcare resource utilization; NICU/ICU, neonatal intensive care unit/intensive care unit; PKU, phenylketonuria; PPY, per person‐year. *p < 0.05.

Rates of inpatient, NICU/ICU, and ED visits were generally low among individuals aged ≥ 12 years, though significantly higher in the PKU cohort compared to controls. Specifically, individuals with PKU had a 39% higher rate of inpatient stays (PKU vs. non‐PKU: 0.44 vs. 0.32 PPY), a 77% higher rate of NICU/ICU stays (0.11 vs. 0.06 PPY), and an 18% higher rate of ED visits (0.94 vs. 0.79 PPY; all *p* < 0.001). In contrast, rates for outpatient visits were substantially higher overall, with individuals aged ≥ 12 years with PKU experiencing a 25% higher rate of outpatient visits compared to controls (15.75 vs. 12.56 PPY; *p* < 0.001) (Figure 3).

Among individuals aged < 12 years, HCRU rates were lower across all settings. Children with PKU had a 23% higher rate of outpatient visits (9.49 vs. 7.75 PPY; *p* < 0.001), but similar rates of inpatient visits (0.07 vs. 0.08 PPY) and NICU/ICU stays (0.02 vs. 0.01 PPY) relative to those without PKU.

##### 3.1.4.2 Costs

**FIGURE 4:**
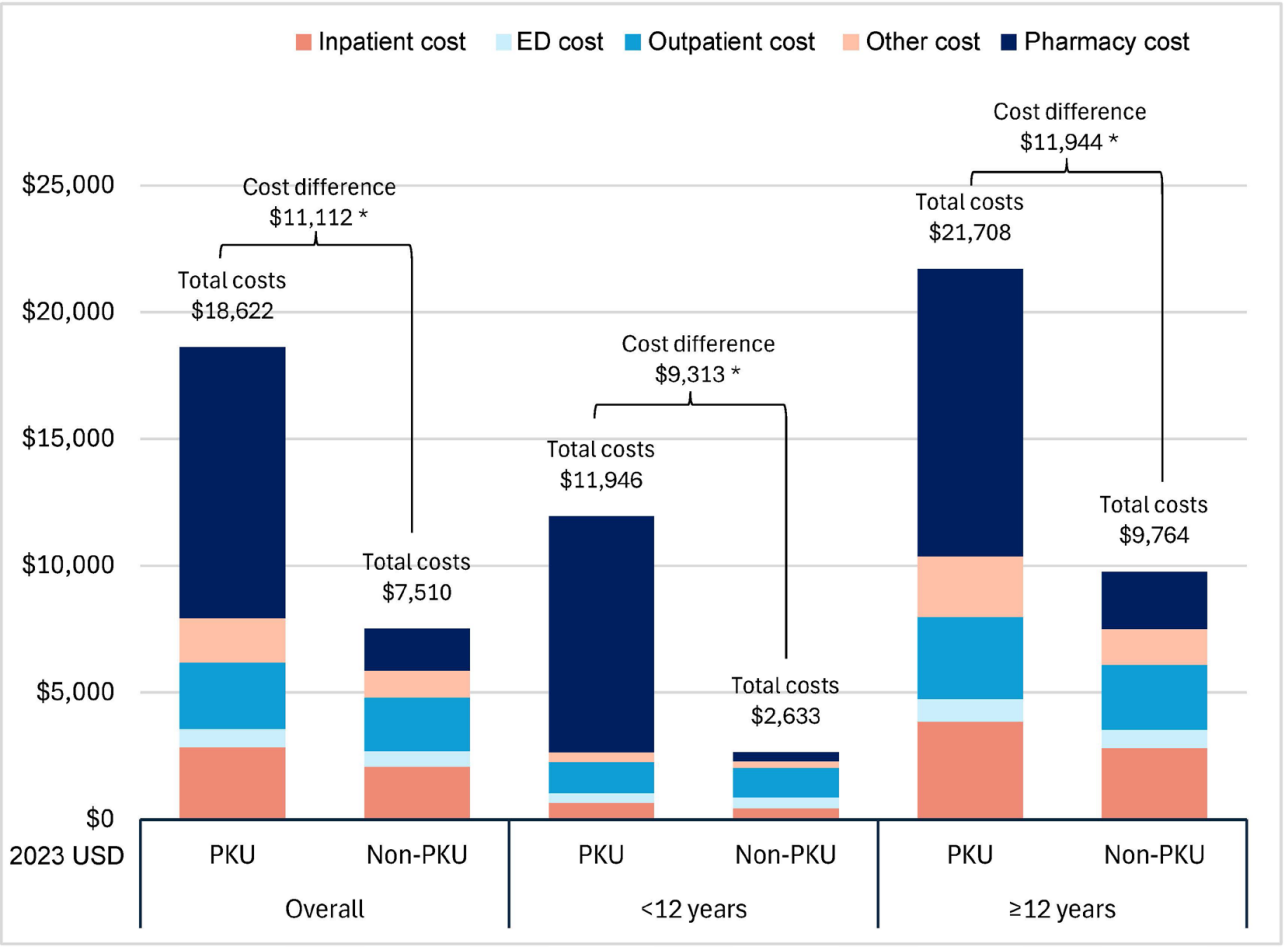
Average annual healthcare costs in individuals with PKU compared to Non‐PKU controls. *Notes:* Healthcare costs represent proxy allowed amounts based on Medicare fee schedules. Encounters with missing cost data and patients with no recorded medical or pharmacy encounters during the observation period were assumed to incur $0 cost. All healthcare costs were inflated to 2023 United States dollars using the annual medical care component of the Consumer Price Index. The date of service was used to link medical costs to the encounter‐level data from the healthcare resource utilization analysis and to flag costs as IP, ED, or OP‐associated. Medical costs that could not be matched to one of these encounter types based on the date of service were classified as “other medical costs.” *p*‐values were calculated using a Wilcoxon signed rank test. Abbreviations: ED, emergency department; PKU, phenylketonuria. *p < 0.05.

Individuals aged ≥ 12 years with PKU had significantly higher total healthcare costs compared to non‐PKU controls. Specifically, the mean annual cost difference was $11 944 ($21 708 for PKU vs. $9764 for non‐PKU; *p* < 0.05), including $9075 in pharmacy costs ($11 346 vs. $2271) and $2869 in medical costs ($10 362 vs. $7493; both *p* < 0.05) (Figure 4, Table S2).

Individuals aged < 12 years with PKU also had significantly higher healthcare costs compared to non‐PKU controls, although the mean annual cost difference was lower than for individuals aged ≥ 12 years. The difference in healthcare costs was $9313 ($11 946 for PKU vs. $2633 for non‐PKU; *p* < 0.05), including $8969 in pharmacy costs ($9310 vs. $341; *p* < 0.05) and only $344 in medical costs ($2636 vs. $2292; not reaching significance) (Figure 4, Table S2). Among individuals with PKU, most pharmacy costs were attributable to the costs of sapropterin dihydrochloride and pegvaliase ($7659 [67.5%] for individuals aged ≥ 12 years and $5594 [60.1%] for individuals aged < 12 years).

### 3.2 Lab Subpopulation

#### 3.2.1 Sample Selection

The lab subpopulation consisted of 403 matched pairs (4.8% of the overall population), with 220 pairs having Phe < 600 μmol/L and 183 pairs having Phe ≥ 600 μmol/L (Figure 1).

#### 3.2.2 Demographic Characteristics

The demographic characteristics of the lab subpopulation are listed in Table 1. Individuals in the lab subpopulation were younger on average (mean [SD]: 18.9 [15.7] years) compared to the overall population, with a higher mean age of 24.7 years for the Phe ≥ 600 μmol/L group compared to 14.1 years for the Phe < 600 μmol/L group.

#### 3.2.3 Clinical Burden

The maximum Phe level and Phe/Tyr ratio were higher on average for individuals with PKU aged ≥ 12 years (mean [SD] 817.6 [558.1] μmol/L and 14.3 [11.1], respectively) compared to those aged < 12 years (480.5 [386.8] μmol/L and 7.5 [9.0], respectively) (Table 2).

**TABLE 2:** Phe and Tyr lab values among individuals with PKU and ≥ 1 Phe lab assessment.

	Lab subpopulation		
	Overall	< 12 years	≥ 12 years
	*N* = 403	*N* = 160	*N* = 243
*Phe lab assessments*			
Number of Phe lab assessments PPY, mean ± SD	1.1 ± 2.4	1.1 ± 1.8	1.2 ± 2.7
Maximum Phe level (μmol/L), mean ± SD	683.7 ± 523.4	480.5 ± 386.8	817.6 ± 558.1
Phe level category (using maximum Phe level)			
< 360 μmol/L	148 (36.7%)	81 (50.6%)	67 (27.6%)
360 to < 600 μmol/L	72 (17.9%)	36 (22.5%)	36 (14.8%)
600 to < 1200 μmol/L	97 (24.1%)	28 (17.5%)	69 (28.4%)
≥ 1200 μmol/L	86 (21.3%)	15 (9.4%)	71 (29.2%)
*Tyr labs*			
Number of patients with Tyr lab assessment(s)	347 (86.1%)	144 (90.0%)	203 (83.5%)
Number of Tyr lab assessments PPY, mean ± SD	1.1 ± 2.6	1.0 ± 1.7	1.1 ± 3.0
Maximum Tyr level (μmol/L), mean ± SD	81.0 ± 57.6	94.9 ± 63.1	71.2 ± 51.2
*Phe to Tyr ratio (using maximum Phe and Tyr levels)*			
Phe to Tyr ratio, mean ± SD	11.5 ± 10.8	7.5 ± 9.0	14.3 ± 11.1
Phe to Tyr ratio ≥ 3	265 (76.4%)	93 (64.6%)	172 (84.7%)

Abbreviations: Phe, phenylalanine; PKU, phenylketonuria; PPY, per‐person year; SD, standard deviation; Tyr, tyrosine.

**FIGURE 5:**
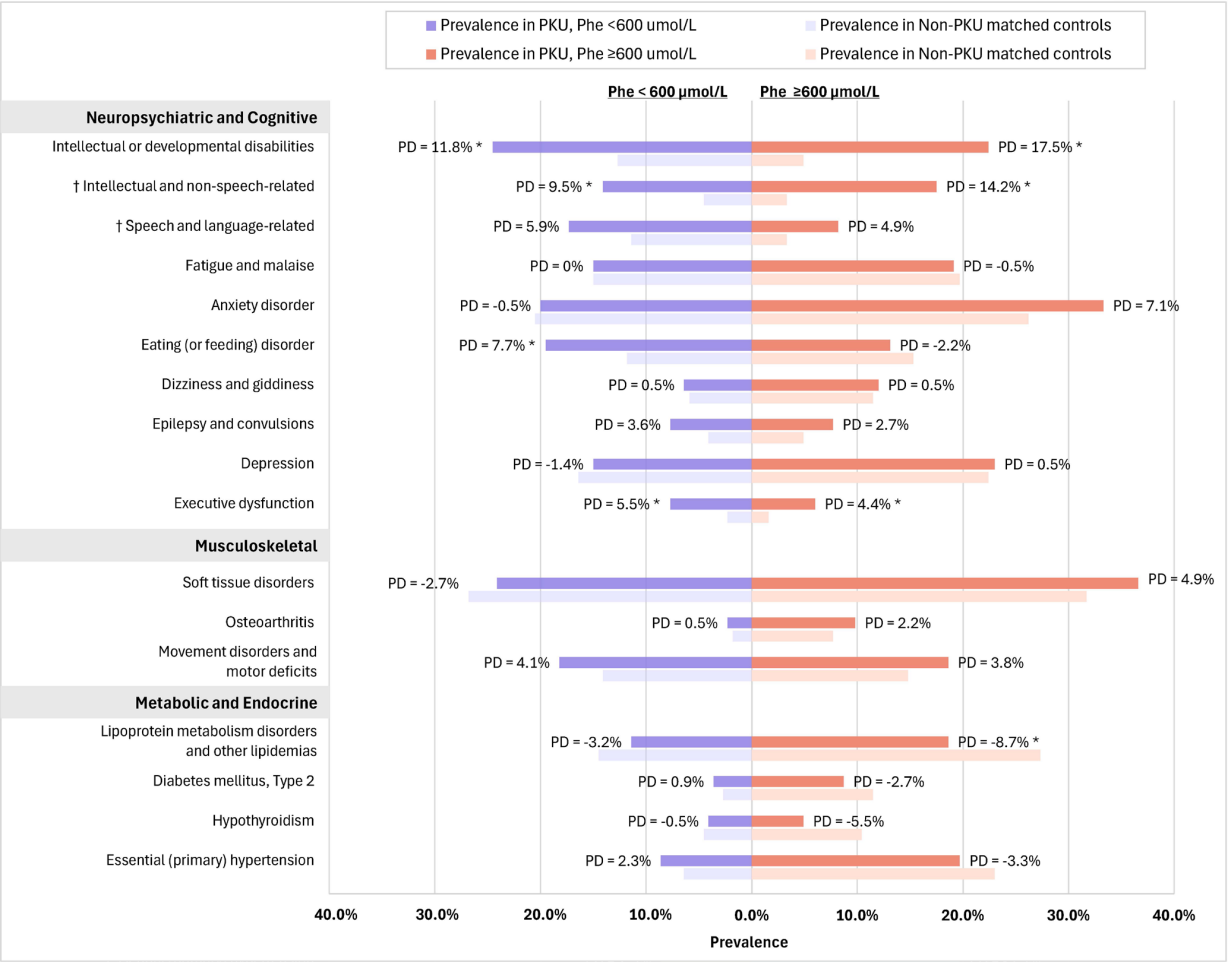
Prevalence of select comorbidities in individuals with PKU compared to Non‐PKU controls, by Phe level. *Note:* Select comorbidities were arranged based on descending order of PD from the overall cohort within each organ system. Among the comprehensive list of PKU‐related comorbidities, the prevalent comorbidities (> 5% in PKU or controls) with a statistically significant difference in the overall population are presented, while the results for the full list of comorbidities are included in the Supplementary Tables. Abbreviations: PD, prevalence difference; Phe, phenylalanine; PKU, phenylketonuria. *p < 0.05. ^†^These comorbidities are subcategories of intellectual or developmental disabilities.

Relative to matched controls without PKU, individuals with PKU had a significantly higher prevalence of IDDs: 24.5% versus 12.7% among those with Phe < 600 μmol/L (PD: 11.8%; *p* < 0.01) and 22.4% versus 4.9% among those with Phe ≥ 600 μmol/L (PD: 17.5%; *p* < 0.001) (Figure 5), with the incremental burden notably higher for individuals with Phe ≥ 600 μmol/L. Executive dysfunction was also significantly more common in those with Phe < 600 and ≥ 600 μmol/L relative to non‐PKU controls (PD: 5.5% and 4.5%, respectively), although the pattern was less clear for other conditions. The results for the full list of comorbidities in the lab subpopulation overall and by Phe level are in Table S3.

#### 3.2.4 Economic Burden

Individuals with PKU had significantly higher total healthcare costs compared to non‐PKU controls among the overall lab subpopulation (mean annual cost difference: $28 473; costs, PKU vs. non‐PKU: $32 978 vs. $4505; *p* < 0.05), mainly driven by the increased total pharmacy costs (Table S5). No clear patterns were observed for HCRU comparing PKU vs. non‐PKU by Phe level (Table S4).

### 3.3 Treatment Subpopulation

Among the overall population, 308 (11.6%) individuals with PKU aged < 12 years and 504 (8.8%) aged ≥ 12 years had ≥ 24 months of follow‐up and received sapropterin dihydrochloride or pegvaliase during follow‐up, therefore qualifying for inclusion in the treatment subpopulation (Figure 1). The mean age was 6.0 (SD: 3.1) years among sapropterin dihydrochloride‐treated individuals with PKU aged < 12 years, and 25.1 (11.4) to 31.7 (10.3) years among those aged ≥ 12 years first treated with sapropterin dihydrochloride and pegvaliase, respectively (Table 3).

**TABLE 3:** Patient characteristics and pharmacological treatment patterns among treated individuals with PKU.

	< 12 years	≥ 12 years	
	Sapropterin dihydrochloride	Sapropterin dihydrochloride	Pegvaliase
	*N* = 308	*N* = 401	*N* = 103
*Patient characteristics*			
Age, mean ± SD (years)	6.0 ± 3.1	25.1 ± 11.4	31.7 ± 10.3
Female, *n* (%)	139 (45.1%)	217 (54.1%)	59 (57.3%)
Medical insurance type, *n* (%)			
Commercial	92 (29.9%)	194 (48.4%)	63 (61.2%)
Medicaid	216 (70.1%)	200 (49.9%)	36 (35.0%)
Medicare Advantage	0 (0.0%)	7 (1.7%)	4 (3.9%)
Index year (2018–2019), *n* (%)	241 (78.2%)	330 (82.3%)	58 (56.3%)
*First treatment*			
Duration of first treatment, mean ± SD (months)	31.4 ± 16.1	19.9 ± 16.0	14.9 ± 13.3
Discontinuation within 18 months^a^, *n* (%)	61 (19.8%)	209 (52.1%)	68 (66.0%)
*Second treatment*			
Initiation of second treatment, *n* (%)	75 (24.4%)	192 (47.9%)	69 (67.0%)
Sapropterin dihydrochloride	72 (23.4%)	101 (25.2%)	8 (7.8%)
Pegvaliase	3 (1.0%)	91 (22.7%)	61 (59.2%)
Time from index to initiation of second treatment, mean ± SD (months)	26.0 ± 13.5	15.9 ± 11.7	13.7 ± 9.8
Initiation of second treatment while on first treatment, among patients with a second treatment, *n* (%)	3 (4.0%)	72 (37.5%)	4 (5.8%)
Initiation of second treatment after discontinuation of first treatment, among patients with a second treatment, *n* (%)	72 (96.0%)	120 (62.5%)	65 (94.2%)

Abbreviations: PKU, phenylketonuria; SD, standard deviation.

^a^ Treatment discontinuation was defined by the initiation of a new treatment from the index (first) treatment or the first gap while on the index treatment. For sapropterin dihydrochloride, an oral drug administered daily, a gap of > 90 days from the last days' supply of the treatment claim was considered discontinuation. For pegvaliase, an injection‐based medication with a recommended dosing regimen of 20 mg once daily during maintenance, a gap of > 30 days from the last days' supply of the treatment claim was considered discontinuation.

Among the pharmacologically treated subpopulation, most individuals aged ≥ 12 years first received sapropterin dihydrochloride (*n* = 401; 79.6%); the remainder (*n* = 103; 20.4%) received pegvaliase as their first treatment. All 308 treated individuals aged < 12 years received sapropterin dihydrochloride since pegvaliase is not approved for those aged < 18 years in the US (Table 3). The mean treatment duration was 31.4 (SD: 16.1) months for individuals aged < 12 years treated with sapropterin dihydrochloride, compared with 14.9 (13.3) and 19.9 (16.0) months for those aged ≥ 12 years first treated with pegvaliase and sapropterin dihydrochloride, respectively. Within 18 months, two‐thirds (*n* = 68; 66.0%) of all treated individuals aged ≥ 12 years discontinued their first observed treatment of pegvaliase, while half (*n* = 209; 52.1%) discontinued their first observed treatment of sapropterin dihydrochloride, compared to only one‐fifth (*n* = 61; 19.8%) of those aged < 12 years who discontinued their first observed treatment of sapropterin dihydrochloride. However, initiation of a second treatment was more commonly observed for individuals aged ≥ 12 years (*n* = 261; 51.8%).

## 4 Discussion

This study leveraged a large US claims database to characterize pharmacological treatment patterns in individuals with PKU and to evaluate the clinical and economic burden of PKU compared to matched controls, overall and by age and blood Phe level. Only 9.7% of individuals with PKU received pharmacological treatment, with older individuals less likely than younger ones to receive drug therapy. Particularly for those aged ≥ 12 years, individuals with PKU had a significantly higher clinical burden compared to controls, which, consistent with prior studies, included neuropsychiatric and cognitive conditions [32, 33] and other comorbidities across multiple organ systems [14, 34, 35].

Among individuals aged ≥ 12 years, the neuropsychiatric and cognitive disorders with the highest incremental burden were IDDs (PD: 5.2%), fatigue and malaise (4.9%), and anxiety disorder (4.2%). These results generally align with the 2017 Bilder et al. study which reported higher rates of IDDs (PD: 4.2%), fatigue and malaise (0.9%), and anxiety disorder (PD: 6.2%) among individuals with PKU compared to matched controls without PKU [32]. Increased prevalence burden for conditions across organ systems was also observed for individuals with PKU in this age group, including but not limited to musculoskeletal (e.g., soft tissue disorders), metabolic and endocrine (e.g., lipoprotein metabolism disorders), cardiorenal (e.g., chronic ischemic heart disease), and digestive (e.g., esophageal disorders) conditions.

Children aged < 12 years with PKU also demonstrated significantly elevated burden for select comorbidities, particularly neuropsychiatric and cognitive conditions such as intellectual and non‐speech‐related developmental disabilities (PD: 2.4%). The early neuropsychiatric and cognitive impacts of PKU are well‐documented, with prior literature reporting deficits in attention and executive function, anxiety and depression, behavioral symptoms, and developmental issues [17, 18, 27, 28, 29, 30, 31, 38, 39]. However, many earlier studies lacked contemporary treatment context, including the use of sapropterin dihydrochloride in pediatric populations. In this analysis, age‐related differences in the clinical burden of PKU were revealed. Adolescents and adults exhibited a broader range of comorbidities and greater incremental burden relative to non‐PKU controls than did young children.

Within the lab subpopulation, lower mean Phe levels were observed among individuals aged < 12 years compared with those ≥ 12 years (480.5 vs. 817.6 μmol/L), consistent with literature reporting increasing Phe levels with age [43, 44]. Given US treatment guidelines recommend maintaining Phe levels < 360 μmol/L [5, 8, 15], results suggest better metabolic control in young children. Further, individuals with elevated Phe levels (≥ 600 μmol/L) demonstrated greater incremental clinical burden compared to controls for several comorbidities—particularly for neuropsychiatric conditions such as IDDs—although interpretation is limited by sparse lab data. Existing real‐world data on PKU burden by Phe level is limited due to the challenges of obtaining lab assessment data. For this study, data from labs outside the Quest network, including State laboratories conducting newborn screening, were unavailable. As a result, the lab subpopulation was small (4.8% of individuals with PKU), and results may not generalize to the broader population with PKU. Phe levels also reflect short‐term metabolic control and fluctuate with diet and treatment, and therefore may not be perfect indicators of disease severity. Individuals in our sample had only one Phe measurement per year on average, limiting longitudinal interpretation and the ability to distinguish pre‐ and post‐treatment levels. Altogether, these constraints may help explain the variability in clinical outcomes observed for the lab subpopulation.

This study also provides valuable insight into the economic burden of PKU in the US, where age‐stratified data on HCRU and costs remain limited. Prior studies such as Trefz et al. [10] have examined international or adult populations, or focused on patient out‐of‐pocket costs [11, 20, 21, 22, 23]. Total healthcare costs among individuals with PKU in this study were found to be 2.2 times those of controls, similar to the 2.3‐fold difference reported by Trefz et al., despite study population and design differences [10]. Higher outpatient utilization among individuals with PKU, also observed by Trefz et al. [10], may reflect the frequent monitoring and care required to manage both PKU and its associated chronic conditions.

Children < 12 years incurred higher healthcare costs than controls, particularly for outpatient visits and pharmacy costs. However, the incremental burden was greatest among adolescents and adults with PKU, aligned with findings from a 2019 US study by Rose et al. [11] showing higher costs in these older age groups.

Despite guidelines recommending lifelong treatment with diet modification and/or medication beginning at birth [5, 8], in this study, only a minority of individuals with PKU received sapropterin dihydrochloride or pegvaliase (8.8% of adolescents and adults; 11.6% of children). Results suggest that dietary management remains the primary treatment approach despite available medications. Among pharmacologically treated individuals with PKU, those aged ≥ 12 years had shorter treatment duration (mean: 14.9–19.9 months vs. 31.4 months) and higher rates of discontinuation within 18 months (52.1%–66.0% vs. 19.8%) than children < 12 years. However, adolescents and adults were also more likely to initiate a second treatment, suggesting that higher discontinuation rates may partly reflect a greater number of available pharmacologic options and more frequent regimen changes for this age group.

Overall, these findings align with prior studies demonstrating stronger Phe control in infancy and childhood and declining control with age [4, 5, 7, 43, 44], attributable to the financial and time demands of maintaining a strict treatment regimen [11, 20, 21, 22, 23], as well as increasing psychosocial challenges with age [45]. The observed pharmacological treatment patterns coupled with heightened clinical and economic burden among individuals ≥ 12 years highlight the substantial unmet needs facing adolescents and adults with PKU.

This study has several notable strengths, including the matching of individuals with PKU to controls without PKU based on key demographic characteristics, which improves comparability and minimizes confounding. The large, closed claims database enabled assessment of comorbidities and estimation of real‐world HCRU and healthcare costs among a diverse US population representing commercial, Medicare Advantage, Medicaid and other insured patients, enhancing study generalizability. Linkage with laboratory data offered additional insight into metabolic control via Phe and Tyr levels and enabled stratified analyses by Phe level. Ultimately, this study provides one of the more comprehensive evaluations of the clinical and economic burden of PKU in individuals < 18 years and contributes rare real‐world data by Phe level.

Our estimates reflect an aggregate PKU population spanning multiple treatment eras, including a subset born before 1965 (pre–widespread newborn screening in the US), for whom comorbidity patterns may differ due to historical differences in diagnosis and treatment. Nonetheless, results were largely consistent in a sensitivity analysis restricted to birth year ≥ 1965, suggesting that the overall conclusions are not driven solely by pre‐1965 birth cohorts.

The limitations of this study include the lack of comprehensive Phe laboratory data, as well as the inherent limitations of retrospective claims database analyses (e.g., miscoding). Because PKU is a genetic disorder and our data captures only a time window within individuals' disease course, initial diagnoses may be missing due to left censoring, limiting evaluation from symptom onset. Similarly, the first observed claim for sapropterin or pegvaliase may not reflect initial treatment. Finally, information on diet therapy, an important component of PKU management, was not available in the claims database. Therefore, treatment patterns for diet therapy, as well as differences in the clinical burden of PKU by adherence to diet therapy, could not be assessed. The economic burden of PKU may be underestimated, as costs of over‐the‐counter medications, special low‐protein foods, and amino acid supplementation were not available. Future studies with robust Phe laboratory data and dietary information are needed to validate the current study's findings and fully address outstanding research gaps in PKU.

## 5 Conclusions

In this large US insurance claims study, adolescents and adults with PKU exhibited significantly higher clinical and economic burdens than matched non‐PKU controls. Among children and individuals with elevated Phe levels, similar—though less pronounced—patterns were observed in the neuropsychiatric and cognitive domains, though interpretation is limited by sample size and sparse laboratory data. Most individuals with PKU, regardless of age, were not receiving pharmacological treatment. Indicators of less controlled disease, including lower treatment adherence and higher Phe levels, were more common among adolescents and adults than young children. Results suggest that the neuropsychiatric and cognitive impacts of PKU may manifest early and expand to additional organ systems over time, leading to a higher overall disease burden with age. The present findings underscore the need for improved PKU treatment strategies, enhanced monitoring, and better adherence support across the lifespan.

## Author Contributions

**Nicola Longo:** conceptualization, methodology, writing – review and editing. **Barbara K. Burton:** conceptualization, methodology, writing – review and editing. **Shailja Vaghela:** conceptualization, funding acquisition, investigation, methodology, project administration, resources, supervision, validation, writing – review and editing. **Fan Mu:** conceptualization, investigation, methodology, project administration, resources, supervision, validation, writing – original draft, writing – review and editing. **Aolin Wang:** conceptualization, investigation, methodology, project administration, resources, supervision, validation, writing – original draft, writing – review and editing. **Jess Smith:** formal analysis, investigation, methodology, project administration, resources, software, supervision, validation, visualization, writing – original draft, writing – review and editing. **Emily Reichert:** formal analysis, investigation, methodology, resources, software, validation, visualization, writing – original draft, writing – review and editing. **Ryan B. Simpson:** conceptualization, investigation, methodology, project administration, validation, writing – original draft, writing – review and editing. **Vanja Sikirica:** conceptualization, funding acquisition, investigation, methodology, resources, supervision, writing – review and editing. **Sue Perera:** conceptualization, funding acquisition, investigation, methodology, project administration, resources, supervision, writing – review and editing. All authors have reviewed and approved the final version of the manuscript for submission. Nicola Longo agrees to serve as the guarantor for the article, accepts full responsibility for the work and/or the conduct of the study, had access to the data, and controlled the decision to publish.

## Funding

This study was supported by Moderna Inc.

## Ethics Statement

The data were de‐identified and comply with requirements of the Health Insurance Portability and Accountability Act of 1996; therefore, no Institutional Review Board waiver of informed consent approval or exemption was required, as per article 45 §CFR 164.514(e).

## Conflicts of Interest

N.L. has served on Advisory Boards and conducts clinical trials with Moderna Inc. S.P. and V.S. are employees of Moderna Inc. and may hold stock or stock options. S.V. is an employee of HealthEcon Consulting Inc. and an external consultant for Moderna Inc. F.M., A.W., J.S., E.R., and R.B.S. are employees of Analysis Group Inc., which has received research funding from Moderna Inc. B.K.B. has received consulting fees and clinical trial funding from Moderna Inc. The authors confirm independence from the sponsors; the content of the article has not been influenced by the sponsors.

## Supporting information


**Table S1:** Prevalence of comorbidities in individuals with PKU compared to Non‐PKU controls
**Table S2:** Average annual healthcare costs in individuals with PKU compared to Non‐PKU controls
**Table S3:** Prevalence of comorbidities in individuals with PKU compared to Non‐PKU controls, by Phe level
**Table S4:** Healthcare resource utilization in individuals with PKU compared to Non‐PKU controls, by Phe level
**Table S5:** Average annual healthcare costs in individuals with PKU compared to Non‐PKU controls, by Phe level

## Data Availability

The data that support the findings of this study are available from HealthVerity. Restrictions apply to the availability of these data, which were used under license for this study.
