# Supplementary material for: Disease Burden and Pharmacological Treatment Patterns in Children and Adults With Phenylketonuria: A Real‐World Matched Cohort Study
Source: J Inherit Metab Dis. 2026 Apr 27;49:e70194. doi: 10.1002/jimd.70194 (PMC13112333; doi:10.1002/jimd.70194)
Supplement: Supplementary file 1 — Table S1: Prevalence of comorbidities in individuals with PKU compared to Non‐PKU controls Table S2: Average annual healthcare costs in individuals with PKU compared to Non‐PKU controls Table S3: Prevalence of comorbidities in individuals with PKU compared to Non‐PKU controls, by Phe level Table S4: Healthcare resource utilization in individuals with PKU compared to Non‐PKU controls, by Phe level Table S5: Average annual healthcare costs in individuals with PKU compared to Non‐PKU controls, by Phe level [file JIMD-49-0-s001.docx]

# SUPPLEMENTAL MATERIALS

**Supplementary Table 1. Prevalence of comorbidities in individuals with PKU compared to Non-PKU controls**

|  | **Overall population (N = 8,377 pairs)** | | | | **<12 years (N = 2,648 pairs)** | | | | **≥12 years (N = 5,729 pairs)** | | | |
| --- | --- | --- | --- | --- | --- | --- | --- | --- | --- | --- | --- | --- |
|  | **PKU** | **Non-PKU** | **PD (%)** | **P-value^a^** | **PKU** | **Non-PKU** | **PD (%)** | **P-value** | **PKU** | **Non-PKU** | **PD (%)** | **P-value^a^** |
| **Follow-up time, months (mean ± SD)** | 35.6 ± 14.3 | 35.6 ± 14.3 | - | - | 33.7 ± 14.0 | 33.7 ± 14.0 | - | - | 36.5 ± 14.3 | 36.5 ± 14.3 | - | - |
| **Neuropsychiatric and Cognitive** |  |  |  |  |  |  |  |  |  |  |  |  |
| Intellectual or developmental disabilities | 1,297 (15.5%) | 957 (11.4%) | 4.1% | <0.001* | 629 (23.8%) | 588 (22.2%) | 1.5% | 0.184 | 668 (11.7%) | 369 (6.4%) | 5.2% | <0.001* |
| Intellectual and non-speech-related developmental disorders | 699 (8.3%) | 343 (4.1%) | 4.2% | <0.001* | 244 (9.2%) | 180 (6.8%) | 2.4% | <0.01* | 455 (7.9%) | 163 (2.8%) | 5.1% | <0.001* |
| Speech and language disorders or delays | 834 (10.0%) | 784 (9.4%) | 0.6% | 0.177 | 540 (20.4%) | 545 (20.6%) | -0.2% | 0.889 | 294 (5.1%) | 239 (4.2%) | 1.0% | <0.05* |
| Fatigue and malaise | 2,155 (25.7%) | 1,884 (22.5%) | 3.2% | <0.001* | 156 (5.9%) | 166 (6.3%) | -0.4% | 0.601 | 1,999 (34.9%) | 1,718 (30.0%) | 4.9% | <0.001* |
| Anxiety disorder | 2,145 (25.6%) | 1,903 (22.7%) | 2.9% | <0.001* | 156 (5.9%) | 154 (5.8%) | 0.1% | 0.951 | 1,989 (34.7%) | 1,749 (30.5%) | 4.2% | <0.001* |
| Eating (or feeding) disorder | 1,265 (15.1%) | 1,041 (12.4%) | 2.7% | <0.001* | 459 (17.3%) | 413 (15.6%) | 1.7% | 0.092 | 806 (14.1%) | 628 (11.0%) | 3.1% | <0.001* |
| Epilepsy and convulsions | 537 (6.4%) | 399 (4.8%) | 1.6% | <0.001* | 119 (4.5%) | 96 (3.6%) | 0.9% | 0.121 | 418 (7.3%) | 303 (5.3%) | 2.0% | <0.001* |
| Depression | 1,755 (21.0%) | 1,629 (19.4%) | 1.5% | <0.01* | 56 (2.1%) | 84 (3.2%) | -1.1% | <0.05* | 1,699 (29.7%) | 1,545 (27.0%) | 2.7% | <0.001* |
| Dizziness and giddiness | 1,147 (13.7%) | 1,028 (12.3%) | 1.4% | <0.01* | 31 (1.2%) | 34 (1.3%) | -0.1% | 0.801 | 1,116 (19.5%) | 994 (17.4%) | 2.1% | <0.01* |
| Dementia | 326 (3.9%) | 240 (2.9%) | 1.0% | <0.001* | 0 (0.0%) | 0 (0.0%) | 0.0% | - | 326 (5.7%) | 240 (4.2%) | 1.5% | <0.001* |
| Mood (affective) disorders | 441 (5.3%) | 359 (4.3%) | 1.0% | <0.01* | 43 (1.6%) | 29 (1.1%) | 0.5% | 0.120 | 398 (6.9%) | 330 (5.8%) | 1.2% | <0.01* |
| Executive dysfunction | 201 (2.4%) | 130 (1.6%) | 0.8% | <0.001* | 50 (1.9%) | 38 (1.4%) | 0.5% | 0.224 | 151 (2.6%) | 92 (1.6%) | 1.0% | <0.001* |
| Autism | 220 (2.6%) | 158 (1.9%) | 0.7% | <0.01* | 134 (5.1%) | 115 (4.3%) | 0.7% | 0.236 | 86 (1.5%) | 43 (0.8%) | 0.8% | <0.001* |
| Behavior and conduct disorders | 262 (3.1%) | 200 (2.4%) | 0.7% | <0.01* | 116 (4.4%) | 110 (4.2%) | 0.2% | 0.733 | 146 (2.5%) | 90 (1.6%) | 1.0% | <0.001* |
| ADHD | 558 (6.7%) | 502 (6.0%) | 0.7% | 0.069 | 228 (8.6%) | 201 (7.6%) | 1.0% | 0.158 | 330 (5.8%) | 301 (5.3%) | 0.5% | 0.245 |
| Sleep disorders | 1,650 (19.7%) | 1,596 (19.1%) | 0.6% | 0.273 | 178 (6.7%) | 219 (8.3%) | -1.5% | <0.05* | 1,472 (25.7%) | 1,377 (24.0%) | 1.7% | <0.05* |
| Cerebral palsy | 83 (1.0%) | 38 (0.5%) | 0.5% | <0.001* | 30 (1.1%) | 14 (0.5%) | 0.6% | <0.05* | 53 (0.9%) | 24 (0.4%) | 0.5% | <0.01* |
| Hemiplegia or paraplegia | 159 (1.9%) | 118 (1.4%) | 0.5% | <0.05* | 12 (0.5%) | 9 (0.3%) | 0.1% | 0.663 | 147 (2.6%) | 109 (1.9%) | 0.7% | <0.05* |
| OCD | 100 (1.2%) | 63 (0.8%) | 0.4% | <0.01* | 11 (0.4%) | 12 (0.5%) | 0.0% | 1.000 | 89 (1.6%) | 51 (0.9%) | 0.7% | <0.01* |
| Microcephaly | 41 (0.5%) | 12 (0.1%) | 0.3% | <0.001* | 25 (0.9%) | 11 (0.4%) | 0.5% | <0.05* | 16 (0.3%) | 1 (0.0%) | 0.3% | <0.001* |
| Migraine and headache | 1,807 (21.6%) | 1,781 (21.3%) | 0.3% | 0.616 | 231 (8.7%) | 251 (9.5%) | -0.8% | 0.340 | 1,576 (27.5%) | 1,530 (26.7%) | 0.8% | 0.325 |
| Tourette’s and Tic disorders | 34 (0.4%) | 18 (0.2%) | 0.2% | <0.05* | 12 (0.5%) | 11 (0.4%) | 0.0% | 1.000 | 22 (0.4%) | 7 (0.1%) | 0.3% | <0.01* |
| Parkinson’s disease | 56 (0.7%) | 43 (0.5%) | 0.2% | 0.228 | 0 (0.0%) | 1 (0.0%) | 0.0% | 1.000 | 56 (1.0%) | 42 (0.7%) | 0.2% | 0.189 |
| Personality disorders | 120 (1.4%) | 103 (1.2%) | 0.2% | 0.275 | 7 (0.3%) | 3 (0.1%) | 0.2% | 0.343 | 113 (2.0%) | 100 (1.7%) | 0.2% | 0.402 |
| Somatoform disorders | 70 (0.8%) | 61 (0.7%) | 0.1% | 0.485 | 3 (0.1%) | 9 (0.3%) | -0.2% | 0.149 | 67 (1.2%) | 52 (0.9%) | 0.3% | 0.199 |
| Schizophrenia and psychosis | 269 (3.2%) | 260 (3.1%) | 0.1% | 0.715 | 3 (0.1%) | 15 (0.6%) | -0.5% | < 0.01* | 266 (4.6%) | 245 (4.3%) | 0.4% | 0.353 |
| Agoraphobia | 22 (0.3%) | 21 (0.3%) | 0.0% | 1.000 | 0 (0.0%) | 0 (0.0%) | 0.0% | - | 22 (0.4%) | 21 (0.4%) | 0.0% | 1.000 |
| Panic disorder | 208 (2.5%) | 206 (2.5%) | 0.0% | 0.960 | 6 (0.2%) | 11 (0.4%) | -0.2% | 0.332 | 202 (3.5%) | 195 (3.4%) | 0.1% | 0.755 |
| Social phobia | 37 (0.4%) | 49 (0.6%) | -0.1% | 0.230 | 4 (0.2%) | 12 (0.5%) | -0.3% | 0.080 | 33 (0.6%) | 37 (0.6%) | -0.1% | 0.716 |
| Bipolar disorder | 320 (3.8%) | 339 (4.0%) | -0.2% | 0.466 | 2 (0.1%) | 9 (0.3%) | -0.3% | 0.070 | 318 (5.6%) | 330 (5.8%) | -0.2% | 0.653 |
| Reaction to severe stress and adjustment disorders | 875 (10.4%) | 914 (10.9%) | -0.5% | 0.328 | 154 (5.8%) | 170 (6.4%) | -0.6% | 0.380 | 721 (12.6%) | 744 (13.0%) | -0.4% | 0.528 |
| Substance abuse | 1,137 (13.6%) | 1,330 (15.9%) | -2.3% | <0.001* | 8 (0.3%) | 17 (0.6%) | -0.3% | 0.110 | 1,129 (19.7%) | 1,313 (22.9%) | -3.2% | <0.001* |
| **Musculoskeletal** |  |  |  |  |  |  |  |  |  |  |  |  |
| Soft tissue disorders | 3,120 (37.2%) | 2,829 (33.8%) | 3.5% | <0.001* | 351 (13.3%) | 348 (13.1%) | 0.1% | 0.933 | 2,769 (48.3%) | 2,481 (43.3%) | 5.0% | <0.001* |
| Osteoarthritis | 1,593 (19.0%) | 1,369 (16.3%) | 2.7% | <0.001* | 2 (0.1%) | 1 (0.0%) | 0.0% | 1.000 | 1,591 (27.8%) | 1,368 (23.9%) | 3.9% | <0.001* |
| Movement disorders and motor deficits | 2,026 (24.2%) | 1,808 (21.6%) | 2.6% | <0.001* | 302 (11.4%) | 292 (11.0%) | 0.4% | 0.693 | 1,724 (30.1%) | 1,516 (26.5%) | 3.6% | <0.001* |
| Movement disorders and motor deficits, other (non-tremor) | 1,914 (22.8%) | 1,692 (20.2%) | 2.7% | <0.001* | 251 (9.5%) | 234 (8.8%) | 0.6% | 0.444 | 1,663 (29.0%) | 1,458 (25.4%) | 3.6% | <0.001* |
| Tremor | 174 (2.1%) | 159 (1.9%) | 0.2% | 0.435 | 14 (0.5%) | 11 (0.4%) | 0.1% | 0.689 | 160 (2.8%) | 148 (2.6%) | 0.2% | 0.523 |
| Osteoporosis | 639 (7.6%) | 507 (6.1%) | 1.6% | <0.001* | 2 (0.1%) | 0 (0.0%) | 0.1% | 0.480 | 637 (11.1%) | 507 (8.8%) | 2.3% | <0.001* |
| Acquired deformities of limbs | 608 (7.3%) | 485 (5.8%) | 1.5% | <0.001* | 99 (3.7%) | 89 (3.4%) | 0.4% | 0.495 | 509 (8.9%) | 396 (6.9%) | 2.0% | <0.001* |
| Dorsopathies, excluding dorsalgia | 1,407 (16.8%) | 1,318 (15.7%) | 1.1% | <0.05* | 75 (2.8%) | 65 (2.5%) | 0.4% | 0.437 | 1,332 (23.3%) | 1,253 (21.9%) | 1.4% | 0.065 |
| Spondylosis | 795 (9.5%) | 747 (8.9%) | 0.6% | 0.175 | 0 (0.0%) | 0 (0.0%) | 0.0% | - | 795 (13.9%) | 747 (13.0%) | 0.8% | 0.175 |
| **Metabolic and Endocrine** |  |  |  |  |  |  |  |  |  |  |  |  |
| Lipoprotein metabolism disorders and other lipidemias | 3,321 (39.6%) | 2,771 (33.1%) | 6.6% | <0.001* | 64 (2.4%) | 80 (3.0%) | -0.6% | 0.192 | 3,257 (56.9%) | 2,691 (47.0%) | 9.9% | <0.001* |
| Diabetes mellitus, Type 2 | 1,974 (23.6%) | 1,464 (17.5%) | 6.1% | <0.001* | 20 (0.8%) | 15 (0.6%) | 0.2% | 0.499 | 1,954 (34.1%) | 1,449 (25.3%) | 8.8% | <0.001* |
| Hypothyroidism | 1,539 (18.4%) | 1,052 (12.6%) | 5.8% | <0.001* | 28 (1.1%) | 17 (0.6%) | 0.4% | 0.136 | 1,511 (26.4%) | 1,035 (18.1%) | 8.3% | <0.001* |
| Essential (primary) hypertension | 2,884 (34.4%) | 2,463 (29.4%) | 5.0% | <0.001* | 23 (0.9%) | 20 (0.8%) | 0.1% | 0.755 | 2,861 (49.9%) | 2,443 (42.6%) | 7.3% | <0.001* |
| Overweight and obesity | 3,453 (41.2%) | 3,260 (38.9%) | 2.3% | <0.001* | 424 (16.0%) | 526 (19.9%) | -3.9% | <0.001* | 3,029 (52.9%) | 2,734 (47.7%) | 5.1% | <0.001* |
| Purine and pyrimidine metabolism disorders | 206 (2.5%) | 60 (0.7%) | 1.7% | <0.001* | 1 (0.0%) | 0 (0.0%) | 0.0% | 1.000 | 205 (3.6%) | 60 (1.0%) | 2.5% | <0.001* |
| Nontoxic goiter | 410 (4.9%) | 302 (3.6%) | 1.3% | <0.001* | 5 (0.2%) | 4 (0.2%) | 0.0% | 1.000 | 405 (7.1%) | 298 (5.2%) | 1.9% | <0.001* |
| Diabetes mellitus, Type 1 | 255 (3.0%) | 158 (1.9%) | 1.2% | <0.001* | 7 (0.3%) | 7 (0.3%) | 0.0% | 1.000 | 248 (4.3%) | 151 (2.6%) | 1.7% | <0.001* |
| Diabetes mellitus, unspecified | 273 (3.3%) | 178 (2.1%) | 1.1% | <0.001* | 0 (0.0%) | 3 (0.1%) | -0.1% | 0.248 | 273 (4.8%) | 175 (3.1%) | 1.7% | <0.001* |
| **Respiratory** |  |  |  |  |  |  |  |  |  |  |  |  |
| Vasomotor and allergic rhinitis | 2,078 (24.8%) | 1,835 (21.9%) | 2.9% | <0.001* | 709 (26.8%) | 742 (28.0%) | -1.2% | 0.304 | 1,369 (23.9%) | 1,093 (19.1%) | 4.8% | <0.001* |
| COPD | 1,070 (12.8%) | 917 (10.9%) | 1.8% | <0.001* | 108 (4.1%) | 84 (3.2%) | 0.9% | 0.090 | 962 (16.8%) | 833 (14.5%) | 2.3% | <0.001* |
| Acute upper respiratory infections | 4,120 (49.2%) | 3,980 (47.5%) | 1.7% | <0.05* | 1,817 (68.6%) | 1,869 (70.6%) | -2.0% | 0.105 | 2,303 (40.2%) | 2,111 (36.8%) | 3.4% | <0.001* |
| Asthma | 1,345 (16.1%) | 1,392 (16.6%) | -0.6% | 0.336 | 392 (14.8%) | 453 (17.1%) | -2.3% | < 0.05 * | 953 (16.6%) | 939 (16.4%) | 0.2% | 0.743 |
| **Cardiorenal** |  |  |  |  |  |  |  |  |  |  |  |  |
| Chronic ischemic heart disease | 1,066 (12.7%) | 688 (8.2%) | 4.5% | <0.001* | 4 (0.2%) | 1 (0.0%) | 0.1% | 0.371 | 1,062 (18.5%) | 687 (12.0%) | 6.5% | <0.001* |
| Atherosclerosis | 897 (10.7%) | 550 (6.6%) | 4.1% | <0.001* | 1 (0.0%) | 1 (0.0%) | 0.0% | 1.000 | 896 (15.6%) | 549 (9.6%) | 6.1% | <0.001* |
| Heart failure | 822 (9.8%) | 537 (6.4%) | 3.4% | <0.001* | 11 (0.4%) | 8 (0.3%) | 0.1% | 0.646 | 811 (14.2%) | 529 (9.2%) | 4.9% | <0.001* |
| CKD | 887 (10.6%) | 678 (8.1%) | 2.5% | <0.001* | 12 (0.5%) | 2 (0.1%) | 0.4% | <0.05* | 875 (15.3%) | 676 (11.8%) | 3.5% | <0.001* |
| Calculus of kidney | 426 (5.1%) | 290 (3.5%) | 1.6% | <0.001* | 4 (0.2%) | 3 (0.1%) | 0.0% | 1.000 | 422 (7.4%) | 287 (5.0%) | 2.4% | <0.001* |
| **Digestive** |  |  |  |  |  |  |  |  |  |  |  |  |
| Esophageal disorders | 2,018 (24.1%) | 1,712 (20.4%) | 3.7% | <0.001* | 119 (4.5%) | 134 (5.1%) | -0.6% | 0.367 | 1,899 (33.1%) | 1,578 (27.5%) | 5.6% | <0.001* |
| Gastroenteritis and colitis | 908 (10.8%) | 740 (8.8%) | 2.0% | <0.001* | 373 (14.1%) | 287 (10.8%) | 3.2% | <0.001* | 535 (9.3%) | 453 (7.9%) | 1.4% | <0.001* |
| Gastritis and duodenitis | 946 (11.3%) | 779 (9.3%) | 2.0% | <0.001* | 55 (2.1%) | 62 (2.3%) | -0.3% | 0.576 | 891 (15.6%) | 717 (12.5%) | 3.0% | <0.001* |
| Gallbladder diseases (gallstones, cholecystitis) | 441 (5.3%) | 325 (3.9%) | 1.4% | <0.001* | 5 (0.2%) | 5 (0.2%) | 0.0% | 1.000 | 436 (7.6%) | 320 (5.6%) | 2.0% | <0.001* |
| **Genitourinary** |  |  |  |  |  |  |  |  |  |  |  |  |
| Urinary system disorders | 2,168 (25.9%) | 1,813 (21.6%) | 4.2% | <0.001* | 218 (8.2%) | 226 (8.5%) | -0.3% | 0.722 | 1,950 (34.0%) | 1,587 (27.7%) | 6.3% | <0.001* |
| Menopausal and other perimenopausal disorders | 614 (7.3%) | 532 (6.4%) | 1.0% | <0.001* | 0 (0.0%) | 0 (0.0%) | 0.0% | - | 614 (10.7%) | 532 (9.3%) | 1.4% | <0.001* |
| Female bleeding disorders | 810 (9.7%) | 794 (9.5%) | 0.2% | 0.640 | 16 (0.6%) | 21 (0.8%) | -0.2% | 0.486 | 794 (13.9%) | 773 (13.5%) | 0.4% | 0.526 |
| Female genital tract noninflammatory disorders | 1,062 (12.7%) | 1,065 (12.7%) | 0.0% | 0.957 | 29 (1.1%) | 52 (2.0%) | -0.9% | < 0.05 * | 1,033 (18.0%) | 1,013 (17.7%) | 0.3% | 0.600 |
| **Dermatological** |  |  |  |  |  |  |  |  |  |  |  |  |
| Eczema and dermatitis | 2,129 (25.4%) | 1,892 (22.6%) | 2.8% | <0.001* | 756 (28.5%) | 801 (30.2%) | -1.7% | 0.165 | 1,373 (24.0%) | 1,091 (19.0%) | 4.9% | <0.001* |
| Melanocytic nevi | 542 (6.5%) | 419 (5.0%) | 1.5% | <0.001* | 28 (1.1%) | 33 (1.2%) | -0.2% | 0.609 | 514 (9.0%) | 386 (6.7%) | 2.2% | <0.001* |
| Alopecia and baldness | 226 (2.7%) | 169 (2.0%) | 0.7% | <0.001* | 18 (0.7%) | 21 (0.8%) | -0.1% | 0.749 | 208 (3.6%) | 148 (2.6%) | 1.0% | <0.01* |
| Urticaria | 331 (4.0%) | 298 (3.6%) | 0.4% | 0.195 | 133 (5.0%) | 152 (5.7%) | -0.7% | 0.272 | 198 (3.5%) | 146 (2.5%) | 0.9% | <0.01* |
| **Other** |  |  |  |  |  |  |  |  |  |  |  |  |
| Anemia | 2,280 (27.2%) | 1,712 (20.4%) | 6.8% | <0.001* | 190 (7.2%) | 255 (9.6%) | -2.5% | <0.01* | 2,090 (36.5%) | 1,457 (25.4%) | 11.0% | <0.001* |
| Abdominal and pelvic pain | 2,839 (33.9%) | 2,663 (31.8%) | 2.1% | <0.01* | 459 (17.3%) | 482 (18.2%) | -0.9% | 0.416 | 2,380 (41.5%) | 2,181 (38.1%) | 3.5% | <0.001* |
| Dorsalgia | 2,504 (29.9%) | 2,417 (28.9%) | 1.0% | 0.093 | 84 (3.2%) | 87 (3.3%) | -0.1% | 0.876 | 2,420 (42.2%) | 2,330 (40.7%) | 1.6% | 0.072 |
| Refraction and accommodation disorders | 2,162 (25.8%) | 2,090 (24.9%) | 0.9% | 0.158 | 390 (14.7%) | 458 (17.3%) | -2.6% | <0.01* | 1,772 (30.9%) | 1,632 (28.5%) | 2.4% | <0.01* |
| Varicose veins of lower extremities | 294 (3.5%) | 228 (2.7%) | 0.8% | <0.01* | 0 (0.0%) | 0 (0.0%) | 0.0% | - | 294 (5.1%) | 228 (4.0%) | 1.2% | <0.01* |
| Family planning or antenatal screening encounter | 962 (11.5%) | 903 (10.8%) | 0.7% | 0.061 | 25 (0.9%) | 19 (0.7%) | 0.2% | 0.429 | 937 (16.4%) | 884 (15.4%) | 0.9% | 0.087 |

**Abbreviations:**ADHD, Attention-deficit/hyperactivity disorder; CKD, chronic kidney disease; COPD, chronic obstructive pulmonary disease; OCD, obsessive compulsive disorder; PD, prevalence difference; PKU, phenylketonuria; SD, standard deviation. * p < 0.05.

**Note:** ^a^ P-values were calculated using a McNemar’s test to assess statistically significant differences in outcomes. Exact p-values were not generated for outcomes with low sample sizes due to the high number of ties.

**Supplementary Table 2. Average annual healthcare costs in individuals with PKU compared to Non-PKU controls**

|  | **Overall population (N = 8,377 pairs)** | | | **<12 years (N = 2,648 pairs)** | | | **≥12 years (N = 5,729 pairs)** | | |
| --- | --- | --- | --- | --- | --- | --- | --- | --- | --- |
|  | **PKU** | **Non-PKU** | **Mean Cost Difference  [PKU - Control]** | **PKU** | **Non-PKU** | **Mean Cost Difference  [PKU - Control]** | **PKU** | **Non-PKU** | **Mean Cost Difference  [PKU - Control]** |
| **Total medical and pharmacy costs, mean ± SD** | $18,622 ± $52,156 | $7,510 ± $25,238 | $11,112 * | $11,946 ± $51,265 | $2,633 ± $6,253 | $9,313 * | $21,708 ± $52,279 | $9,764 ± $29,955 | $11,944 * |
| **Total medical costs, mean ± SD** | $7,920 ± $33,848 | $5,849 ± $23,166 | $2,071 * | $2,636 ± $7,970 | $2,292 ± $5,057 | $344 | $10,362 ± $40,338 | $7,493 ± $27,647 | $2,869 * |
| Inpatient costs | $2,837 ± $14,749 | $2,065 ± $10,334 | $772 * | $660 ± $5,214 | $450 ± $2,890 | $210 | $3,843 ± $17,388 | $2,811 ± $12,270 | $1,032 * |
| ED costs | $731 ± $3,142 | $629 ± $1,681 | $102 | $370 ± $771 | $419 ± $712 | -$49 * | $897 ± $3,752 | $726 ± $1,966 | $171 * |
| Outpatient costs | $2,607 ± $5,272 | $2,111 ± $3,839 | $496 * | $1,216 ± $2,265 | $1,158 ± $2,142 | $58 * | $3,249 ± $6,079 | $2,552 ± $4,337 | $697 * |
| Other costs | $1,745 ± $27,007 | $1,044 ± $18,259 | $701 * | $390 ± $3,790 | $265 ± $1,938 | $125 * | $2,372 ± $32,537 | $1,404 ± $22,031 | $968 * |
| **Total pharmacy costs, mean ± SD** | $10,702 ± $39,029 | $1,661 ± $7,280 | $9,041 * | $9,310 ± $50,059 | $342 ± $2,332 | $8,968 * | $11,346 ± $32,681 | $2,271 ± $8,591 | $9,075 * |

**Abbreviations:** ED, emergency department; PKU, phenylketonuria; SD, standard deviation. *p < 0.05.

**Notes:** ^a^ Healthcare costs represent proxy allowed amounts based on Medicare fee schedules. Encounters with missing cost data, and patients with no recorded medical or pharmacy encounters during the observation period, were assumed to incur $0 cost. All healthcare costs were inflated to 2023 U.S. dollars (USD) using the annual medical care component of the Consumer Price Index (CPI). ^b^ The date of service was used to link medical costs to the encounter-level data from the healthcare resource utilization (HCRU) analysis and to flag costs as IP, ED, or OP-associated. Medical costs that could not be matched to one of these encounter types based on the date of service were classified as ‘other medical costs’. ^c^ P-values were calculated using a Wilcoxon signed rank test.

**Supplementary Table 3. Prevalence of comorbidities in individuals with PKU compared to Non-PKU controls, by Phe level**

|  | **Lab subpopulation**  **(N=43 pairs)** | | | | **Phe <600 µmol/L (N = 220 pairs)** | | | | **Phe ≥600 µmol/L (N = 183 pairs)** | | | |
| --- | --- | --- | --- | --- | --- | --- | --- | --- | --- | --- | --- | --- |
|  | **PKU** | **Non-PKU** | **PD (%)** | **P-value^a^** | **PKU** | **Non-PKU** | **PD (%)** | **P-value** | **PKU** | **Non-PKU** | **PD (%)** | **P-value^a^** |
| **Follow-up time, months (mean ± SD)** | 40.0 ± 13.9 | 40.0 ± 13.9 | - | - | 39.2 ± 13.7 | 39.2 ± 13.7 | - | - | 40.9 ± 14.1 | 40.9 ± 14.1 | - | - |
| **Neuropsychiatric and Cognitive** |  |  |  |  |  |  |  |  |  |  |  |  |
| Intellectual or developmental disabilities | 95 (23.6%) | 37 (9.2%) | 14.4% | < 0.001 * | 54 (24.5%) | 28 (12.7%) | 11.8% | < 0.01 * | 41 (22.4%) | 9 (4.9%) | 17.5% | < 0.001 * |
| Intellectual and non-speech-related developmental disorders | 63 (15.6%) | 16 (4.0%) | 11.7% | < 0.001 * | 31 (14.1%) | 10 (4.5%) | 9.5% | < 0.01 * | 32 (17.5%) | 6 (3.3%) | 14.2% | < 0.001 * |
| Speech and language disorders or delays | 53 (13.2%) | 31 (7.7%) | 5.5% | < 0.01 * | 38 (17.3%) | 25 (11.4%) | 5.9% | 0.080 | 15 (8.2%) | 6 (3.3%) | 4.9% | 0.052 |
| Fatigue and malaise | 68 (16.9%) | 69 (17.1%) | -0.2% | 1.000 | 33 (15.0%) | 33 (15.0%) | 0.0% | 1.000 | 35 (19.1%) | 36 (19.7%) | -0.5% | 1.000 |
| Anxiety disorder | 105 (26.1%) | 93 (23.1%) | 3.0% | 0.323 | 44 (20.0%) | 45 (20.5%) | -0.5% | 1.000 | 61 (33.3%) | 48 (26.2%) | 7.1% | 0.124 |
| Eating (or feeding) disorder | 67 (16.6%) | 54 (13.4%) | 3.2% | 0.237 | 43 (19.5%) | 26 (11.8%) | 7.7% | < 0.05 * | 24 (13.1%) | 28 (15.3%) | -2.2% | 0.651 |
| Epilepsy and convulsions | 31 (7.7%) | 18 (4.5%) | 3.2% | 0.067 | 17 (7.7%) | 9 (4.1%) | 3.6% | 0.118 | 14 (7.7%) | 9 (4.9%) | 2.7% | 0.404 |
| Depression | 75 (18.6%) | 77 (19.1%) | -0.5% | 0.920 | 33 (15.0%) | 36 (16.4%) | -1.4% | 0.766 | 42 (23.0%) | 41 (22.4%) | 0.5% | 1.000 |
| Dizziness and giddiness | 36 (8.9%) | 34 (8.4%) | 0.5% | 0.897 | 14 (6.4%) | 13 (5.9%) | 0.5% | 1.000 | 22 (12.0%) | 21 (11.5%) | 0.5% | 1.000 |
| Dementia | 3 (0.7%) | 1 (0.2%) | 0.5% | 0.480 | 1 (0.5%) | 0 (0.0%) | 0.5% | 1.000 | 2 (1.1%) | 1 (0.5%) | 0.5% | 1.000 |
| Mood (affective) disorders | 30 (7.4%) | 12 (3.0%) | 4.5% | < 0.01 * | 14 (6.4%) | 5 (2.3%) | 4.1% | 0.066 | 16 (8.7%) | 7 (3.8%) | 4.9% | 0.095 |
| Executive dysfunction | 28 (6.9%) | 8 (2.0%) | 5.0% | < 0.001 * | 17 (7.7%) | 5 (2.3%) | 5.5% | < 0.01 * | 11 (6.0%) | 3 (1.6%) | 4.4% | < 0.05 * |
| Autism | 22 (5.5%) | 5 (1.2%) | 4.2% | < 0.01 * | 16 (7.3%) | 3 (1.4%) | 5.9% | < 0.01 * | 6 (3.3%) | 2 (1.1%) | 2.2% | 0.289 |
| Behavior and conduct disorders | 27 (6.7%) | 19 (4.7%) | 2.0% | 0.268 | 16 (7.3%) | 10 (4.5%) | 2.7% | 0.307 | 11 (6.0%) | 9 (4.9%) | 1.1% | 0.803 |
| ADHD | 57 (14.1%) | 44 (10.9%) | 3.2% | 0.198 | 33 (15.0%) | 26 (11.8%) | 3.2% | 0.381 | 24 (13.1%) | 18 (9.8%) | 3.3% | 0.429 |
| Sleep disorders | 56 (13.9%) | 61 (15.1%) | -1.2% | 0.668 | 24 (10.9%) | 28 (12.7%) | -1.8% | 0.643 | 32 (17.5%) | 33 (18.0%) | -0.5% | 0.010 |
| Cerebral palsy | 5 (1.2%) | 2 (0.5%) | 0.7% | 0.450 | 3 (1.4%) | 0 (0.0%) | 1.4% | 0.248 | 2 (1.1%) | 2 (1.1%) | 0.0% | 1.000 |
| Hemiplegia or paraplegia | 5 (1.2%) | 3 (0.7%) | 0.5% | 0.683 | 3 (1.4%) | 2 (0.9%) | 0.5% | 1.000 | 2 (1.1%) | 1 (0.5%) | 0.5% | 1.000 |
| OCD | 3 (0.7%) | 4 (1.0%) | -0.2% | 1.000 | 0 (0.0%) | 2 (0.9%) | -0.9% | 0.480 | 3 (1.6%) | 2 (1.1%) | 0.5% | 0.010 |
| Microcephaly | 1 (0.2%) | 0 (0.0%) | 0.2% | 1.000 | 1 (0.5%) | 0 (0.0%) | 0.5% | 1.000 | 0 (0.0%) | 0 (0.0%) | 0.0% | - |
| Migraine and headache | 86 (21.3%) | 112 (27.8%) | -6.5% | < 0.05 * | 42 (19.1%) | 54 (24.5%) | -5.5% | 0.169 | 44 (24.0%) | 58 (31.7%) | -7.7% | 0.110 |
| Tourette’s and Tic disorders | 4 (1.0%) | 2 (0.5%) | 0.5% | 0.683 | 2 (0.9%) | 2 (0.9%) | 0.0% | 1.000 | 2 (1.1%) | 0 (0.0%) | 1.1% | 0.480 |
| Parkinson’s disease | 0 (0.0%) | 2 (0.5%) | -0.5% | 0.480 | 0 (0.0%) | 1 (0.5%) | -0.5% | 1.000 | 0 (0.0%) | 1 (0.5%) | -0.5% | 1.000 |
| Personality disorders | 5 (1.2%) | 5 (1.2%) | 0.0% | 1.000 | 2 (0.9%) | 3 (1.4%) | -0.5% | 1.000 | 3 (1.6%) | 2 (1.1%) | 0.5% | 1.000 |
| Somatoform disorders | 1 (0.2%) | 0 (0.0%) | 0.2% | 1.000 | 1 (0.5%) | 0 (0.0%) | 0.5% | 1.000 | 0 (0.0%) | 0 (0.0%) | 0.0% | - |
| Schizophrenia and psychosis | 8 (2.0%) | 17 (4.2%) | -2.2% | 0.110 | 2 (0.9%) | 7 (3.2%) | -2.3% | 0.182 | 6 (3.3%) | 10 (5.5%) | -2.2% | 0.453 |
| Agoraphobia | 2 (0.5%) | 3 (0.7%) | -0.2% | 1.000 | 0 (0.0%) | 0 (0.0%) | 0.0% | - | 2 (1.1%) | 3 (1.6%) | -0.5% | 1.000 |
| Panic disorder | 8 (2.0%) | 12 (3.0%) | -1.0% | 0.502 | 0 (0.0%) | 6 (2.7%) | -2.7% | < 0.05 * | 8 (4.4%) | 6 (3.3%) | 1.1% | 0.789 |
| Social phobia | 6 (1.5%) | 4 (1.0%) | 0.5% | 0.752 | 2 (0.9%) | 3 (1.4%) | -0.5% | 1.000 | 4 (2.2%) | 1 (0.5%) | 1.6% | 0.371 |
| Bipolar disorder | 10 (2.5%) | 17 (4.2%) | -1.7% | 0.248 | 5 (2.3%) | 5 (2.3%) | 0.0% | 1.000 | 5 (2.7%) | 12 (6.6%) | -3.8% | 0.146 |
| Reaction to severe stress and adjustment disorders | 51 (12.7%) | 47 (11.7%) | 1.0% | 0.749 | 22 (10.0%) | 26 (11.8%) | -1.8% | 0.643 | 29 (15.8%) | 21 (11.5%) | 4.4% | 0.302 |
| Substance abuse | 31 (7.7%) | 51 (12.7%) | -5.0% | < 0.05 * | 15 (6.8%) | 19 (8.6%) | -1.8% | 0.556 | 16 (8.7%) | 32 (17.5%) | -8.7% | < 0.05 * |
| **Musculoskeletal** |  |  |  |  |  |  |  |  |  |  |  |  |
| Soft tissue disorders | 120 (29.8%) | 117 (29.0%) | 0.7% | 0.861 | 53 (24.1%) | 59 (26.8%) | -2.7% | 0.550 | 67 (36.6%) | 58 (31.7%) | 4.9% | 0.306 |
| Osteoarthritis | 23 (5.7%) | 18 (4.5%) | 1.2% | 0.458 | 5 (2.3%) | 4 (1.8%) | 0.5% | 1.000 | 18 (9.8%) | 14 (7.7%) | 2.2% | 0.540 |
| Movement disorders and motor deficits | 74 (18.4%) | 58 (14.4%) | 4.0% | 0.153 | 40 (18.2%) | 31 (14.1%) | 4.1% | 0.313 | 34 (18.6%) | 27 (14.8%) | 3.8% | 0.381 |
| Movement disorders and motor deficits, other (non-tremor) | 69 (17.1%) | 57 (14.1%) | 3.0% | 0.281 | 36 (16.4%) | 30 (13.6%) | 2.7% | 0.511 | 33 (18.0%) | 27 (14.8%) | 3.3% | 0.461 |
| Tremor | 10 (2.5%) | 2 (0.5%) | 2.0% | < 0.05 * | 6 (2.7%) | 0 (0.0%) | 2.7% | < 0.05 * | 4 (2.2%) | 2 (1.1%) | 1.1% | 0.683 |
| Osteoporosis | 11 (2.7%) | 3 (0.7%) | 2.0% | < 0.05 * | 4 (1.8%) | 0 (0.0%) | 1.8% | 0.134 | 7 (3.8%) | 3 (1.6%) | 2.2% | 0.289 |
| Acquired deformities of limbs | 12 (3.0%) | 18 (4.5%) | -1.5% | 0.345 | 7 (3.2%) | 12 (5.5%) | -2.3% | 0.332 | 5 (2.7%) | 6 (3.3%) | -0.5% | 1.000 |
| Dorsopathies, excluding dorsalgia | 50 (12.4%) | 40 (9.9%) | 2.5% | 0.289 | 27 (12.3%) | 19 (8.6%) | 3.6% | 0.243 | 23 (12.6%) | 21 (11.5%) | 1.1% | 0.868 |
| Spondylosis | 18 (4.5%) | 14 (3.5%) | 1.0% | 0.584 | 5 (2.3%) | 4 (1.8%) | 0.5% | 1.000 | 13 (7.1%) | 10 (5.5%) | 1.6% | 0.663 |
| **Metabolic and Endocrine** |  |  |  |  |  |  |  |  |  |  |  |  |
| Lipoprotein metabolism disorders and other lipidemias | 59 (14.6%) | 82 (20.3%) | -5.7% | < 0.05 * | 25 (11.4%) | 32 (14.5%) | -3.2% | 0.360 | 34 (18.6%) | 50 (27.3%) | -8.7% | < 0.05 * |
| Diabetes mellitus, Type 2 | 24 (6.0%) | 27 (6.7%) | -0.7% | 0.749 | 8 (3.6%) | 6 (2.7%) | 0.9% | 0.752 | 16 (8.7%) | 21 (11.5%) | -2.7% | 0.458 |
| Hypothyroidism | 18 (4.5%) | 29 (7.2%) | -2.7% | 0.109 | 9 (4.1%) | 10 (4.5%) | -0.5% | 1.000 | 9 (4.9%) | 19 (10.4%) | -5.5% | 0.066 |
| Essential (primary) hypertension | 55 (13.6%) | 56 (13.9%) | -0.2% | 1.000 | 19 (8.6%) | 14 (6.4%) | 2.3% | 0.404 | 36 (19.7%) | 42 (23.0%) | -3.3% | 0.377 |
| Overweight and obesity | 127 (31.5%) | 156 (38.7%) | -7.2% | < 0.05 * | 55 (25.0%) | 81 (36.8%) | -11.8% | < 0.01 * | 72 (39.3%) | 75 (41.0%) | -1.6% | 0.826 |
| Purine and pyrimidine metabolism disorders | 1 (0.2%) | 0 (0.0%) | 0.2% | 1.000 | 1 (0.5%) | 0 (0.0%) | 0.5% | 1.000 | 0 (0.0%) | 0 (0.0%) | 0.0% | - |
| Nontoxic goiter | 7 (1.7%) | 11 (2.7%) | -1.0% | 0.453 | 4 (1.8%) | 2 (0.9%) | 0.9% | 0.683 | 3 (1.6%) | 9 (4.9%) | -3.3% | 0.114 |
| Diabetes mellitus, Type 1 | 2 (0.5%) | 2 (0.5%) | 0.0% | 1.000 | 1 (0.5%) | 1 (0.5%) | 0.0% | 1.000 | 1 (0.5%) | 1 (0.5%) | 0.0% | 1.000 |
| Diabetes mellitus, unspecified | 4 (1.0%) | 2 (0.5%) | 0.5% | 0.683 | 2 (0.9%) | 1 (0.5%) | 0.5% | 1.000 | 2 (1.1%) | 1 (0.5%) | 0.5% | 1.000 |
| **Respiratory** |  |  |  |  |  |  |  |  |  |  |  |  |
| Vasomotor and allergic rhinitis | 106 (26.3%) | 114 (28.3%) | -2.0% | 0.582 | 56 (25.5%) | 66 (30.0%) | -4.5% | 0.343 | 50 (27.3%) | 48 (26.2%) | 1.1% | 0.907 |
| COPD | 23 (5.7%) | 20 (5.0%) | 0.7% | 0.749 | 6 (2.7%) | 11 (5.0%) | -2.3% | 0.332 | 17 (9.3%) | 9 (4.9%) | 4.4% | 0.136 |
| Acute upper respiratory infections | 219 (54.3%) | 210 (52.1%) | 2.2% | 0.526 | 132 (60.0%) | 121 (55.0%) | 5.0% | 0.278 | 87 (47.5%) | 89 (48.6%) | -1.1% | 0.907 |
| Asthma | 57 (14.1%) | 83 (20.6%) | -6.5% | < 0.05 * | 28 (12.7%) | 49 (22.3%) | -9.5% | < 0.05 * | 29 (15.8%) | 34 (18.6%) | -2.7% | 0.568 |
| **Cardiorenal** |  |  |  |  |  |  |  |  |  |  |  |  |
| Chronic ischemic heart disease | 7 (1.7%) | 6 (1.5%) | 0.2% | 1.000 | 0 (0.0%) | 1 (0.5%) | -0.5% | 1.000 | 7 (3.8%) | 5 (2.7%) | 1.1% | 0.773 |
| Atherosclerosis | 4 (1.0%) | 5 (1.2%) | -0.2% | 1.000 | 1 (0.5%) | 1 (0.5%) | 0.0% | 1.000 | 3 (1.6%) | 4 (2.2%) | -0.5% | 1.000 |
| Heart failure | 8 (2.0%) | 6 (1.5%) | 0.5% | 0.752 | 3 (1.4%) | 0 (0.0%) | 1.4% | 0.248 | 5 (2.7%) | 6 (3.3%) | -0.5% | 1.000 |
| CKD | 5 (1.2%) | 4 (1.0%) | 0.2% | 1.000 | 2 (0.9%) | 0 (0.0%) | 0.9% | 0.480 | 3 (1.6%) | 4 (2.2%) | -0.5% | 1.000 |
| Calculus of kidney | 12 (3.0%) | 6 (1.5%) | 1.5% | 0.239 | 3 (1.4%) | 1 (0.5%) | 0.9% | 0.617 | 9 (4.9%) | 5 (2.7%) | 2.2% | 0.423 |
| **Digestive** |  |  |  |  |  |  |  |  |  |  |  |  |
| Esophageal disorders | 55 (13.6%) | 51 (12.7%) | 1.0% | 0.720 | 21 (9.5%) | 16 (7.3%) | 2.3% | 0.458 | 34 (18.6%) | 35 (19.1%) | -0.5% | 1.000 |
| Gastroenteritis and colitis | 30 (7.4%) | 32 (7.9%) | -0.5% | 0.894 | 17 (7.7%) | 20 (9.1%) | -1.4% | 0.728 | 13 (7.1%) | 12 (6.6%) | 0.5% | 1.000 |
| Gastritis and duodenitis | 21 (5.2%) | 23 (5.7%) | -0.5% | 0.877 | 7 (3.2%) | 9 (4.1%) | -0.9% | 0.803 | 14 (7.7%) | 14 (7.7%) | 0.0% | 1.000 |
| Gallbladder diseases (gallstones, cholecystitis) | 10 (2.5%) | 8 (2.0%) | 0.5% | 0.814 | 4 (1.8%) | 5 (2.3%) | -0.5% | 1.000 | 6 (3.3%) | 3 (1.6%) | 1.6% | 0.505 |
| **Genitourinary** |  |  |  |  |  |  |  |  |  |  |  |  |
| Urinary system disorders | 75 (18.6%) | 68 (16.9%) | 1.7% | 0.569 | 37 (16.8%) | 35 (15.9%) | 0.9% | 0.892 | 38 (20.8%) | 33 (18.0%) | 2.7% | 0.596 |
| Menopausal and other perimenopausal disorders | 9 (2.2%) | 8 (2.0%) | 0.2% | 1.000 | 2 (0.9%) | 3 (1.4%) | -0.5% | 1.000 | 7 (3.8%) | 5 (2.7%) | 1.1% | 0.724 |
| Female bleeding disorders | 40 (9.9%) | 44 (10.9%) | -1.0% | 0.671 | 21 (9.5%) | 19 (8.6%) | 0.9% | 0.845 | 19 (10.4%) | 25 (13.7%) | -3.3% | 0.307 |
| Female genital tract noninflammatory disorders | 53 (13.2%) | 54 (13.4%) | -0.2% | 1.000 | 28 (12.7%) | 24 (10.9%) | 1.8% | 0.584 | 25 (13.7%) | 30 (16.4%) | -2.7% | 0.499 |
| **Dermatological** |  |  |  |  |  |  |  |  |  |  |  |  |
| Eczema and dermatitis | 85 (21.1%) | 96 (23.8%) | -2.7% | 0.379 | 41 (18.6%) | 52 (23.6%) | -5.0% | 0.215 | 44 (24.0%) | 44 (24.0%) | 0.0% | 1.000 |
| Melanocytic nevi | 36 (8.9%) | 13 (3.2%) | 5.7% | < 0.01 * | 18 (8.2%) | 5 (2.3%) | 5.9% | < 0.05 * | 18 (9.8%) | 8 (4.4%) | 5.5% | 0.055 |
| Alopecia and baldness | 7 (1.7%) | 8 (2.0%) | -0.2% | 1.000 | 5 (2.3%) | 3 (1.4%) | 0.9% | 0.724 | 2 (1.1%) | 5 (2.7%) | -1.6% | 0.450 |
| Urticaria | 9 (2.2%) | 17 (4.2%) | -2.0% | 0.170 | 3 (1.4%) | 12 (5.5%) | -4.1% | < 0.05 * | 6 (3.3%) | 5 (2.7%) | 0.5% | 1.000 |
| **Other** |  |  |  |  |  |  |  |  |  |  |  |  |
| Anemia | 52 (12.9%) | 51 (12.7%) | 0.2% | 1.000 | 24 (10.9%) | 26 (11.8%) | -0.9% | 0.871 | 28 (15.3%) | 25 (13.7%) | 1.6% | 0.755 |
| Abdominal and pelvic pain | 109 (27.0%) | 134 (33.3%) | -6.2% | 0.065 | 54 (24.5%) | 70 (31.8%) | -7.3% | 0.122 | 55 (30.1%) | 64 (35.0%) | -4.9% | 0.356 |
| Dorsalgia | 95 (23.6%) | 91 (22.6%) | 1.0% | 0.781 | 46 (20.9%) | 43 (19.5%) | 1.4% | 0.791 | 49 (26.8%) | 48 (26.2%) | 0.5% | 1.000 |
| Refraction and accommodation disorders | 105 (26.1%) | 104 (25.8%) | 0.2% | 1.000 | 59 (26.8%) | 59 (26.8%) | 0.0% | 1.000 | 46 (25.1%) | 45 (24.6%) | 0.5% | 1.000 |
| Varicose veins of lower extremities | 4 (1.0%) | 5 (1.2%) | -0.2% | 1.000 | 1 (0.5%) | 2 (0.9%) | -0.5% | 1.000 | 3 (1.6%) | 3 (1.6%) | 0.0% | 1.000 |
| Family planning or antenatal screening encounter | 60 (14.9%) | 64 (15.9%) | -1.0% | 0.708 | 29 (13.2%) | 29 (13.2%) | 0.0% | 1.000 | 31 (16.9%) | 35 (19.1%) | -2.2% | 0.607 |

**Abbreviations:**ADHD, attention-deficit/hyperactivity disorder; CKD, chronic kidney disease; COPD, chronic obstructive pulmonary disease; OCD, obsessive compulsive disorder; PD, prevalence difference; Phe, phenylalanine; PKU, phenylketonuria; SD, standard deviation. * p<0.05.

**Note:** ^a^ P-values were calculated using a McNemar’s test to assess statistically significant differences in outcomes. Exact p-values were not generated for outcomes with low sample sizes due to the high number of ties.

**Supplementary Table 4. Healthcare resource utilization in individuals with PKU compared to Non-PKU controls, by Phe level**

| **All-cause HCRU, PPY, Rate ratio (95% CI)** | **Lab subpopulation** | **Phe <600 µmol/L** | **Phe ≥600 µmol/L** |
| --- | --- | --- | --- |
|  | **N = 403 pairs** | **N = 220 pairs** | **N = 183 pairs** |
| Inpatient visits | 1.19 (0.67, 2.11) | 1.13 (0.50, 2.54) | 1.26 (0.57, 2.77) |
| NICU/ICU visits | 3.12 (1.26, 7.76) * | 2.17 (0.53, 8.96) | 4.01 (1.25, 12.88) * |
| ED visits | 0.98 (0.80, 1.21) | 0.84 (0.66, 1.07) | 1.16 (0.82, 1.64) |
| Outpatient visits | 1.24 (1.05, 1.46) * | 1.26 (1.02, 1.57) * | 1.22 (0.95, 1.57) |

**Abbreviations:** CI, confidence interval; ED, emergency department; HCRU, healthcare resource utilization; NICU/ICU, neonatal intensive care unit/intensive care unit; Phe, phenylalanine; PKU, phenylketonuria; PPY, per person-year. * p<0.05.

**Notes:** ^a^ Rate ratios and 95% CIs were calculated using a generalized estimating equations model with data clustered by matching ID. The model used a log link function (outcome was assumed to be Poisson distributed), with an exchangeable correlation structure. ^b^ HCRU settings were flagged at the day level based on place of service codes, type of bill codes, revenue codes, and Current Procedural Terminology/Healthcare Common Procedure Coding System codes. Encounter periods were created based on the presence of flags in consecutive days, except for OP stays for which each day was counted as an individual visit. ED and outpatient stays that overlapped with an inpatient visit were removed. ^c^ Four ED stays were removed from the data due to long length of stays greater than 100 days.

**Supplementary Table 5. Average annual healthcare costs in individuals with PKU compared to Non-PKU controls, by Phe level**

|  | **Lab subpopulation (N = 403 pairs)** | | | **Phe <600 µmol/L (N = 220 pairs)** | | | **Phe ≥600 µmol/L (N = 183 pairs)** | | |
| --- | --- | --- | --- | --- | --- | --- | --- | --- | --- |
|  | **PKU** | **Non-PKU** | **Mean Cost Difference  [PKU - Control]** | **PKU** | **Non-PKU** | **Mean Cost Difference  [PKU - Control]** | **PKU** | **Non-PKU** | **Mean Cost Difference  [PKU - Control]** |
| **Total medical and pharmacy costs, mean ± SD** | $32,978 ± $71,152 | $4,505 ± $8,901 | $28,473 * | $31,239 ± $80,182 | $3,578 ± $5,536 | $27,661 * | $35,068 ± $58,635 | $5,620 ± $11,654 | $29,448 * |
| **Total medical costs, mean ± SD** | $4,385 ± $9,626 | $3,578 ± $7,123 | $807 | $4,284 ± $10,418 | $2,994 ± $4,474 | $1,290 | $4,506 ± $8,604 | $4,280 ± $9,331 | $226 |
| Inpatient costs | $1,345 ± $5,687 | $939 ± $3,443 | $406 | $1,150 ± $5,404 | $738 ± $2,478 | $412 | $1,579 ± $6,017 | $1,182 ± $4,323 | $397 |
| ED costs | $625 ± $1,328 | $622 ± $1,741 | $3 | $479 ± $885 | $574 ± $1,151 | -$95 | $800 ± $1,702 | $680 ± $2,256 | $120 |
| Outpatient costs | $1,756 ± $2,667 | $1,613 ± $2,879 | $143 * | $1,706 ± $2,230 | $1,548 ± $3,012 | $158 * | $1,816 ± $3,117 | $1,691 ± $2,716 | $125 |
| Other costs | $659 ± $5,422 | $404 ± $2,983 | $255 * | $949 ± $7,305 | $135 ± $496 | $814 * | $311 ± $710 | $727 ± $4,377 | -$416 * |
| **Total pharmacy costs, mean ± SD** | $28,593 ± $69,805 | $927 ± $3,298 | $27,666 * | $26,955 ± $78,320 | $584 ± $2,194 | $26,371 * | $30,562 ± $58,091 | $1,340 ± $4,232 | $29,222 * |

**Abbreviations:** ED, emergency department; Phe, phenylalanine; PKU, phenylketonuria; SD, standard deviation. * p<0.05

**Notes:** ^a^ Healthcare costs represent proxy allowed amounts based on Medicare fee schedules. Encounters with missing cost data, and patients with no recorded medical or pharmacy encounters during the observation period, were assumed to incur $0 cost. All healthcare costs were inflated to 2023 United States dollars using the annual medical care component of the Consumer Price Index. ^b^ The date of service was used to link medical costs to the encounter-level data from the healthcare resource utilization analysis and to flag costs as inpatient, ED, or outpatient-associated. Medical costs that could not be matched to one of these encounter types based on the date of service were classified as ‘other medical costs’. ^c^ P-values were calculated using a Wilcoxon signed rank test.
